# Reclassification of *Botryococcus braunii* chemical races into separate species based on a comparative genomics analysis

**DOI:** 10.1371/journal.pone.0304144

**Published:** 2024-07-29

**Authors:** Devon J. Boland, Ivette Cornejo-Corona, Daniel R. Browne, Rebecca L. Murphy, John Mullet, Shigeru Okada, Timothy P. Devarenne

**Affiliations:** 1 Department of Biochemistry and Biophysics, Texas A & M University, College Station, Texas, United States of America; 2 Texas A&M Institute for Genome Sciences & Society (TIGSS), College Station, Texas, United States of America; 3 AI & Computational Biology, LanzaTech Inc., Skokie, Illinois, United States of America; 4 Biology Department, Centenary College of Louisiana, Shreveport, Louisiana, United States of America; 5 Laboratory of Aquatic Natural Products Chemistry, Graduate School of Agricultural and Life Sciences, The University of Tokyo, Yayoi, Bunkyo, Tokyo, Japan; Central University of Kerala, INDIA

## Abstract

The colonial green microalga *Botryococcus braunii* is well known for producing liquid hydrocarbons that can be utilized as biofuel feedstocks. *B*. *braunii* is taxonomically classified as a single species made up of three chemical races, A, B, and L, that are mainly distinguished by the hydrocarbons produced. We previously reported a B race draft nuclear genome, and here we report the draft nuclear genomes for the A and L races. A comparative genomic study of the three *B*. *braunii* races and 14 other algal species within *Chlorophyta* revealed significant differences in the genomes of each race of *B*. *braunii*. Phylogenomically, there was a clear divergence of the three races with the A race diverging earlier than both the B and L races, and the B and L races diverging from a later common ancestor not shared by the A race. DNA repeat content analysis suggested the B race had more repeat content than the A or L races. Orthogroup analysis revealed the *B*. *braunii* races displayed more gene orthogroup diversity than three closely related *Chlamydomonas* species, with nearly 24-36% of all genes in each *B*. *braunii* race being specific to each race. This analysis suggests the three races are distinct species based on sufficient differences in their respective genomes. We propose reclassification of the three chemical races to the following species names: *Botryococcus alkenealis* (A race), *Botryococcus braunii* (B race), and *Botryococcus lycopadienor* (L race).

## Introduction

*Botryococcus braunii* (Trebouxiophyceae) is a colonial green microalga that has long been studied due to its ability to biosynthesize large quantities of liquid hydrocarbons with potential use as biofuel feedstocks [1, 2]. Purified hydrocarbons from *B*. *braunii* have been refined into petroleum-fuel equivalents to provide so-called “drop-in” fuels that can be used with the current combustive fuel infrastructure [3–5]. *B*. *braunii* is further split into three distinct chemical races based on the hydrocarbons produced in each race: The odd carbon numbered C_23-33_ alkadiene/alkatriene producing A race, the C_30-37_ botryococcene and C_31-34_ methylsqualene producing B race, and the C_40_ lycopadiene producing L race [6–8]. More recently, a potential fourth S chemical race was isolated with the major hydrocarbons identified as shorter chain saturated C_18_ epoxy-*n*-alkanes or a C_20_ *n*-alkane [9]. This study focuses on the A, B, and L races of *B*. *braunii*.

Historically, studies on *B*. *braunii* have focused on hydrocarbon biosynthesis, cell morphology, extracellular matrix (ECM) composition, and phylogenetics [1, 10–21]. For the hydrocarbons, the A race alkenes are derived from fatty acids utilizing C_18_ oleic acid as a precursor [10, 11, 13], while the B and L races utilize the isoprenoid-derived molecules C_15_ farnesyl diphosphate and C_20_ geranylgeranyl diphosphate, respectively, as precursors for hydrocarbon production [22]. Morphologically, the cell sizes differ between the races with the L race displaying smaller sizes (8-9μm x 5μm) than the A or B races (13μm x 7-9 μm) [12]. As a consequence of the different hydrocarbons, each race has a distinct ECM composition with the hydrocarbons in each race linked by epoxide bridges to long-chain polyacetals spanning the ECM. Phylogeny studies focused on the 18S rRNA gene revealed distinct clade-level groupings for each of the three races [9, 16, 17, 20, 23]. Despite these many differences, *B*. *braunii* is taxonomically classified as a single species made up of distinct races consisting of various geographic strains within each race.

One traditional definition for species classification of organisms utilizes the biological species concept, in which organisms are defined as separate species if they cannot cross via sexual reproduction [24]. Chlorophyta contains species that reproduce sexually or asexually [25, 26]. For example, *Chlamydomonas reinhardtii* and its close relatives reproduce sexually through the regulation of two key genes that determine gamete mating type [27, 28]. These sexual *Chlamydomonas* reproduction-relevant genes have homologs in closely related species such as those within the *Volvox* genus [29, 30]. *B*. *braunii* has not been observed to reproduce sexually and is assumed to reproduce only asexually [20]. Since the conventional method for determining species classification cannot be applied, analysis of genome-wide characteristics may offer insights into the question of speciation among the *B*. *braunii* races.

Comparative genomic studies have been used as evidence for the classification of species. Recently, the genomes of three redpoll bird species in the genus *Acanthus* were used to reclassify these individual species as a single species using a linked supergene that controls phenotypic color differences [31]. Additionally, it is common practice to utilize comparative genomic analysis when classifying newly discovered bacteria [32, 33]. With the increased accuracy of long-read sequencing and improvement of assembly algorithms for organisms with complex, repeat-rich genomes such as plants, a comparative genomic study of *B*. *braunii* would aid in the current understanding of the organism and the relationships between the three races. Recently, we published a draft nuclear genome assembly for the B race, Berkeley (or Showa) strain of *B*. *braunii* [34]. For the current study, we sequenced and assembled draft nuclear genomes for the A and L races and performed a comparative genomic analysis of *B*. *braunii* at the race and larger taxonomic levels. This analysis revealed significant differences at the genomic level between the three *B*. *braunii* races that we propose are significant enough to reclassify the races as separate species.

## Results

### *Botryococcus braunii* genome assemblies

High quality, high molecular weight genomic DNA [34, 35] was isolated from the A, B, and L races, and this DNA was used for sequencing on the Oxford Nanopore Technologies. This long-read genomic sequencing data was combined with previously obtained Illumina short-read sequencing for all three chemical races to combine the benefits of both sequencing technologies for downstream genome assembly. This produced 18-24x coverage (three samples multiplexed and sequenced on two flow cells) from the Nanopore data and 140-316x coverage from the Illumina data. Coverage was calculated based on total bases from sequencing divided by the estimated genome size for each race [16, 17]. For each race, *de novo*, reference-guided, and hybrid (combing Oxford Nanopore and Illumina sequences) assemblies were tested to determine which method produced an optimal draft genome assembly based on assembly metrics and BUSCO scores. For the L race, a *de novo* assembly approach using only the Oxford Nanopore sequence data produced the best assembly. For the A race, a hybrid assembly method, combining both Oxford Nanopore and Illumina sequence data, produced the most contiguous genome assembly. All attempts to produce an improved genome assembly for the B race resulted in sub-par final assemblies and were less contiguous compared to the current B race genome assembly [34].

The genome assembly statistics for three green microalgae with relatively complete assemblies, *Chlamydomonas reinhardtii* [36], *Coccomyxa subellipsoidea* [37], and *Chlorella variabilis* [38], are included in Table 1 as references to evaluate the contiguousness of the new *B*. *braunii* A and L race genome assemblies. Of the three *B*. *braunii* race genome assemblies, the L race was the most contiguous and whole with the largest N50 value and the smallest number of contigs/scaffolds, meaning it is less fragmented overall (Table 1). Even though the A race had a similar number of contigs/scaffolds as the B race genome assembly, its L50 was nearly half that of the B race assembly (Table 1), meaning the longest scaffolds assembled for the A race are longer and more contiguous than those of the B race. All three of the *B*. *braunii* race genome assemblies had a low percentage of fragmented BUSCOs while having varying amounts of single-copy, complete BUSCOs (Table 1). Overall, the new *B*. *braunii* A and L race genome assemblies are more contiguous than the current B race genome assembly. Interestingly the L race had the smallest assembly size of all three races at 135.6 Mb, and the A race had the largest at 188.3 Mb (Table 1). This contradicts earlier genome size estimates that showed the L race had the largest genome size at 211 Mb followed by the B race at 166 Mb and the A race at 160 Mb [16, 17]. Due to the difference in assembled genome sizes versus earlier estimations, all three race assemblies would benefit from additional sequencing including long-read and Hi-C sequencing in hopes of anchoring scaffolds to achieve chromosome resolution genome assemblies. This marks the first time that all three *B*. *braunii* races have had genomes assembled.

**Table 1:** Assembly statistics for all three chemical races of *B*. *braunii*. Assembly statistics for all three chemical races of *B*. *braunii*. A and L chemical races are novel in this study. The *B*. *braunii* B race, *C*. *reinhardtii*, *C*. *variabilis*, and *C*. *subellipsoidea* serve as a comparison to assess the contiguity of the A and L race genomes. Both A and L race assemblies are at scaffold resolution containing a mix of scaffolds and contigs. BUSCO scores were generated using the Chlorophyta ODBv10.

Species	*B*. *braunii*, race A	*B*. *braunii*, r*ace B*	*B*. *Braunii*, r*ace L*	*Chlamydomonas reinhardtii*	*Coccomyxa subellipsoidea*	*Chlorella* *variabilis*
**Assembly Size (Mb)**	**188.3**	**170.2**	**135.6**	**111.1**	**48.9**	**46.2**
**Number of unitigs** **(scaffolds/contigs)**	^**a**^ **903** **(897/28)**	**983**	^**a**^ **485** **(8/477)**	**54**	**45**	**414**
**N50 (Mb)**	^**b**^ **0.752** **(0.375/2.93)**	**0.565**	^**b**^ **2.91** **(3.94/2.64)**	**7.78**	**1.96**	**1.47**
**L50**	^**b**^ **47** **(85/9)**	**99**	^**b**^ **15** **(3/13)**	**7**	**9**	**12**
**GC Content (%)**	**49.98**	**50.82**	**52.51**	**64.08**	**52.93**	**67.14**
^**c**^ **BUSCO Score** **(% Complete/** **% Fragmented)**	**83.9/0.6**	**89.6/1.8**	**90.7/0.8**	**96.5/1.7**	**98.5/0.4**	**95.7/2.0**
^**d**^ **BUSCO Score (% Complete/** **% Fragmented)**	**71.7/1.11**	**70.4/5.33**	**83.1/0.65**	**97.9/0.32**	**95.8/0.85**	**91.9/2.56**

^a^: The top number indicates number of unitigs (scaffolds plus contigs), first number in parentheses indicates number of scaffolds, second number indicates number of contigs.

^b^: For N50 and L50 metrics, the top number indicates the metrics calculated using combined scaffolds and contigs, first number in parentheses indicates metrics calculated using only scaffolds, second number only contigs.

^c^- BUSCO run in “genome” mode.

^d^- BUSCO run in “protein” mode.

### Genome sizes vary among Trebouxiophyceae

Our early estimations of *B*. *braunii* genome sizes had placed the A, B, and L races among the largest known genomes for green microalgae at the time [16, 17]. Since these studies, more Chlorophyta species have been sequenced and genomes assembled, annotated, and deposited in public databases such as PhycoCosm [39]. This has allowed for a reexamination of *B*. *braunii* genome sizes and how they compare to other green microalgae.

For this comparison, we obtained genome size information from PhycoCosm for 14 species of green microalgae from the Trebouxiophyceae and Chlorophyceae clades as shown in S1 Table in S1 File. These species were chosen because of their close relation to *B*. *braunii* to serve as an in-clade comparison (Trebouxiophyceae) or as an outlier group for external clade comparison (Chlorophyceae). Additional criteria for comparison selection were datasets that included masked genomes, assembled transcriptomes, and annotated gene sets (gtf/gff3 formatted files). The comparison of the *B*. *braunii* genomes to these 14 species of green microalgae showed that the *B*. *braunii* genome assemblies are the largest among the Trebouxiophyceae species analyzed, but not the largest when including the Chlorophyceae species, with the *Dunaliella salina* genome [40] being the largest of those analyzed (S1 Table in S1 File). The closest known relative to *B*. *braunii*, *C*. *subellipsoidea*, had a substantially smaller (48.9Mb) genome assembly size [37] compared to any of the three *B*. *braunii* genome assemblies (Table 1 and S1 Table in S1 File). This large difference in genome assembly size would suggest that after divergence from a common ancestor either *B*. *braunii* experienced a genome size expansion event(s) or *C*. *subellipsoidea* experienced a genome size reduction event. The genome assembly sizes for the 9 species of Trebouxiophyceae (not including *B*. *braunii)* analyzed, are in the range of 14-60Mb (S1 Table in S1 File), suggesting *B*. *braunii* underwent a genome size expansion after delineation from a common ancestor shared with *C*. *subellipsoidea*.

**Fig 1:**
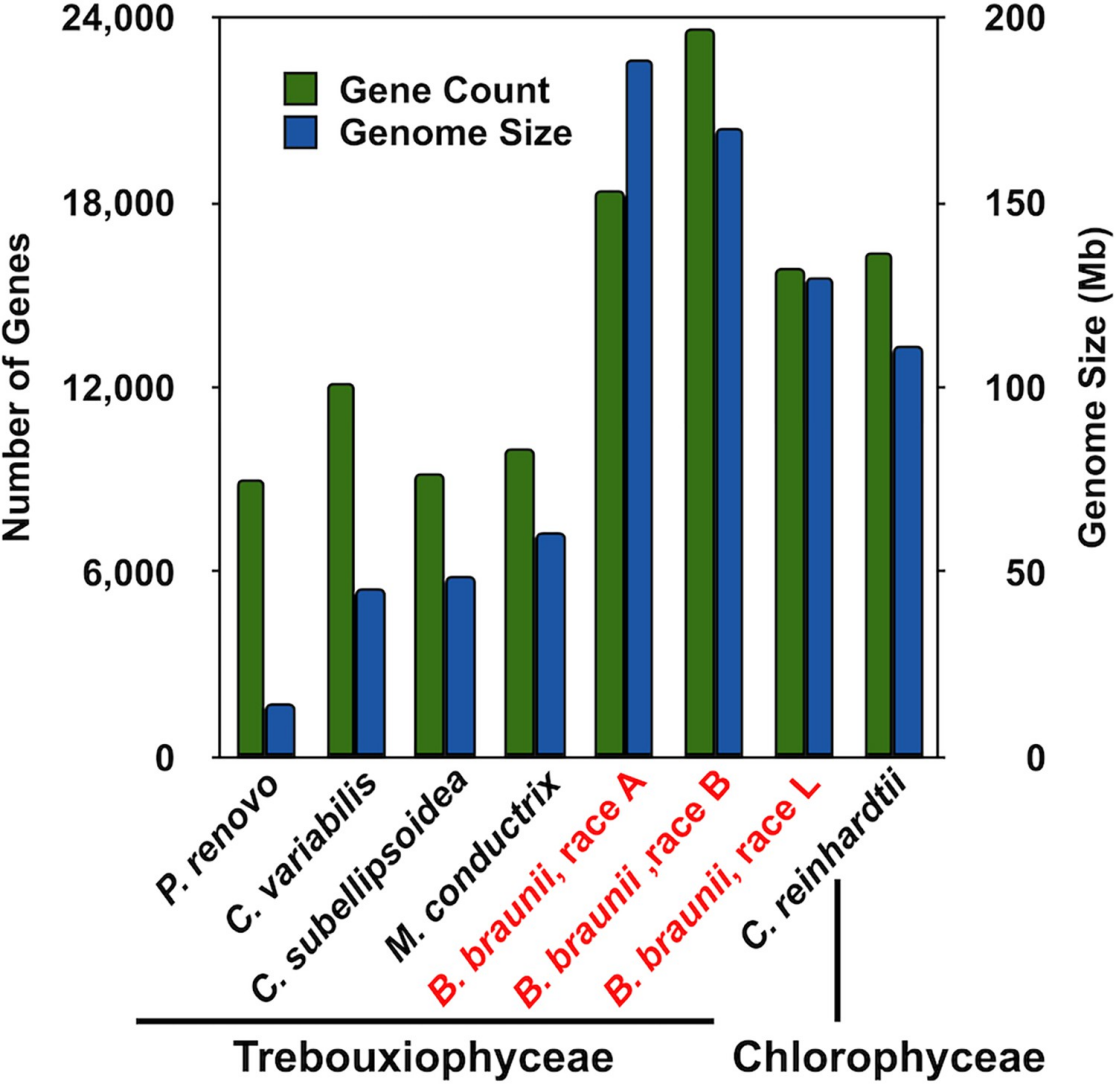
Comparison of gene number and genome size for select Chlorophyta species. Grouped bar chart showing genome assembly size in blue and predicted gene count for each assembly in green. All algae except *C*. *reinhardtii* are Trebouxiophyceae. *C*. *reinhardtii* is shown as a reference for a similar size and gene count compared to the *B*. *braunii* races.

It has been hypothesized that the cause of the large *B*. *braunii* genome sizes was a large number of genes encoded in the genomes of each race [16, 17]. This seemed to be confirmed when we published the first *B*. *braunii* genome from the B race [34], which had 20,765 genes. Now with the A and L race genomes assemblies, this finding can be reanalyzed by comparing genome size and gene count in several Trebouxiophyceae species and *C*. *reinhardtii* from Chlorophyceae. In general, an increase in genome size correlates with an increase in gene count for most Trebouxiophyceae species analyzed (Fig 1 and S1 Table in S1 File). It should be clarified that in this work the number of genes reported as encoded on the B race genome, 23,685, differs from the published value of 20,765 [34]. This is because the annotation software used to perform gene annotation in the A and L races was also applied to the B race genome (see Methods section for detailed description), due to this software’s increased capabilities over what was used when the B race genome was published. Genome size and gene count are not positively correlated among the three races of *B*. *braunii*. The *B*. *braunii* L race has a ~90Mb larger genome assembly size compared to *C*. *variabilis* and has ~6,000 more genes. (Fig 1 and S1 Table in S1 File). Similarly, *B*. *braunii* A race has a ~140Mb larger genome assembly size compared to *C*. *variabilis* and ~9,000 more genes. Within *B*. *braunii*, the A race, with the largest *B*. *braunii* genome assembly size, has ~5,200 fewer and ~2,600 more genes than the B and L races, respectively (Fig 1). Additionally, gene coding sequences in each race were analyzed for overlap with repeat content (see below for description of repeat content). This analysis revealed that only the A race contained gene/repeat overlaps with 3,189 genes having a significant overlap (>70%) to repeat elements (S1 File). Interestingly, *Picochlorum renovo* displays the smallest known Trebouxiophyceae genome size, yet has a remarkably large number of encoded genes given the small genome size (Fig 1) [41]. This compact genome may be a consequence of the fitness this species displays in thermo, salinity, and intense light tolerance [42–45]. Since gene count did not appear to be the sole factor accounting for the large *B*. *braunii* assembled genome sizes observed, a more detailed investigation into the genomes of *B*. *braunii* was carried out.

### Gene feature distribution of *B*. *braunii*

**Fig 2:**
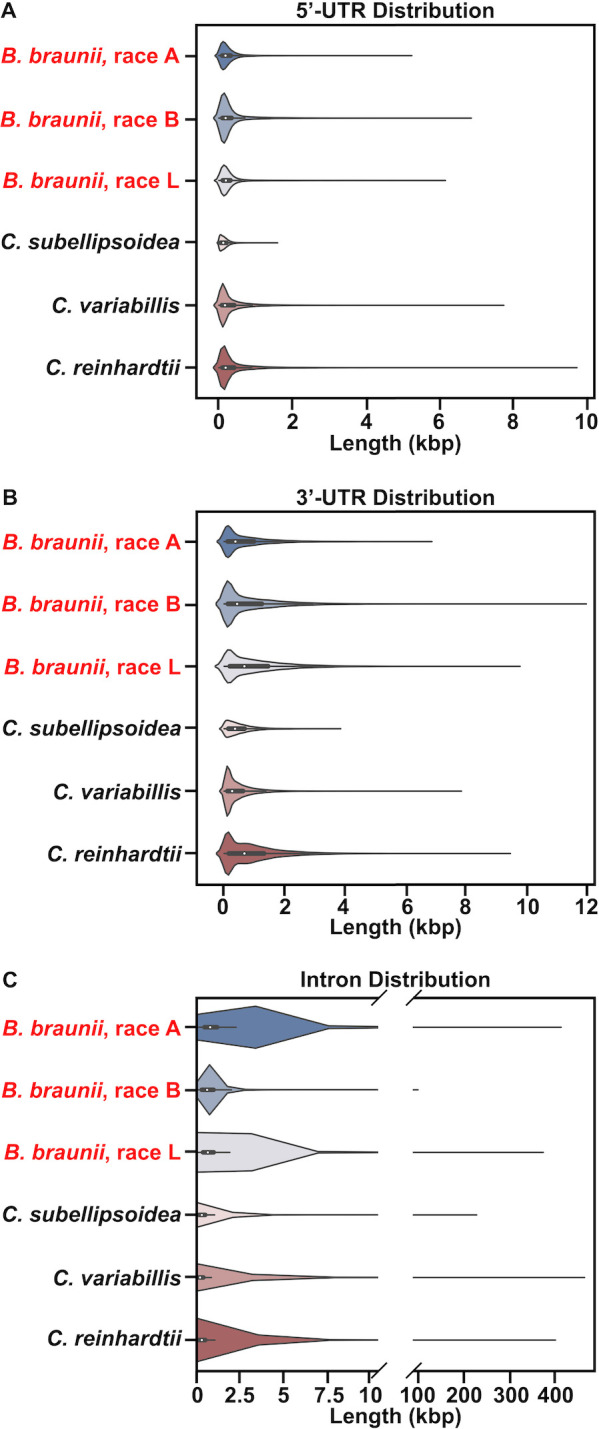
Non-coding gene feature distribution analysis. Box plots inside violin plots for (A) 5’-untranslated regions (UTRs), (B) 3’-untranslated regions (UTR)s, and (C) Intron lengths in the three races of *B*. *braunii*, *C*. *subellipsoidea*, *C*. *variabilis*, and *C*. *reinhardtii*. Box plots indicate length distribution with the median gene feature length indicated by a white dot inside the box plot. Violin plots show the frequency (width of the curve) of gene features at a given length (x-axis).

Having genome assemblies for the first time for each race of *B*. *braunii* allowed for direct comparison of gene feature distributions such as the length of 5’-UTRs, 3’-UTRs, and introns. These *B*. *braunii* gene features were compared to two Trebouxiophyceae species, *C*. *variabilis* and *C*. *subellipsoidea*, and the Chlorophyceae species *C*. *reinhardtii*. These species were chosen as a representative sample of different levels of relation to *B*. *braunii* given our phylogenomic analysis (see Fig 4): *C*. *subellipsoidea* as the closest relative, *C*. *variabilis* as a more distant relative, and *C*. *reinhardtii* as a distant relative form a different clade. The distribution of 5’- and 3’-UTRs was similar for each species analyzed with the largest number of UTR regions in each species being between ~100 and ~500 bp (Fig 2A and 2B). However, differences between species can be seen in the small number of large UTRs seen in each species. For example, among the three *B*. *braunii* races the B race has the longest 5’-UTRs and 3’-UTRs (Fig 2A and 2B). Interestingly, the closely related *C*. *subellipsoidea* had the smallest number of and shortest length of both types of UTRs (Fig 2A and 2B). For intron length distribution, the B race of *B*. *braunii* had a smaller number of and shorter length of introns compared to the A and L races (Fig 2C). This phenomenon could explain the large number of genes encoded in the B race genome assembly, but may also be an artifact of its current highly fragmented state.

### DNA repeat content of *B*. *braunii*

**Fig 3:**
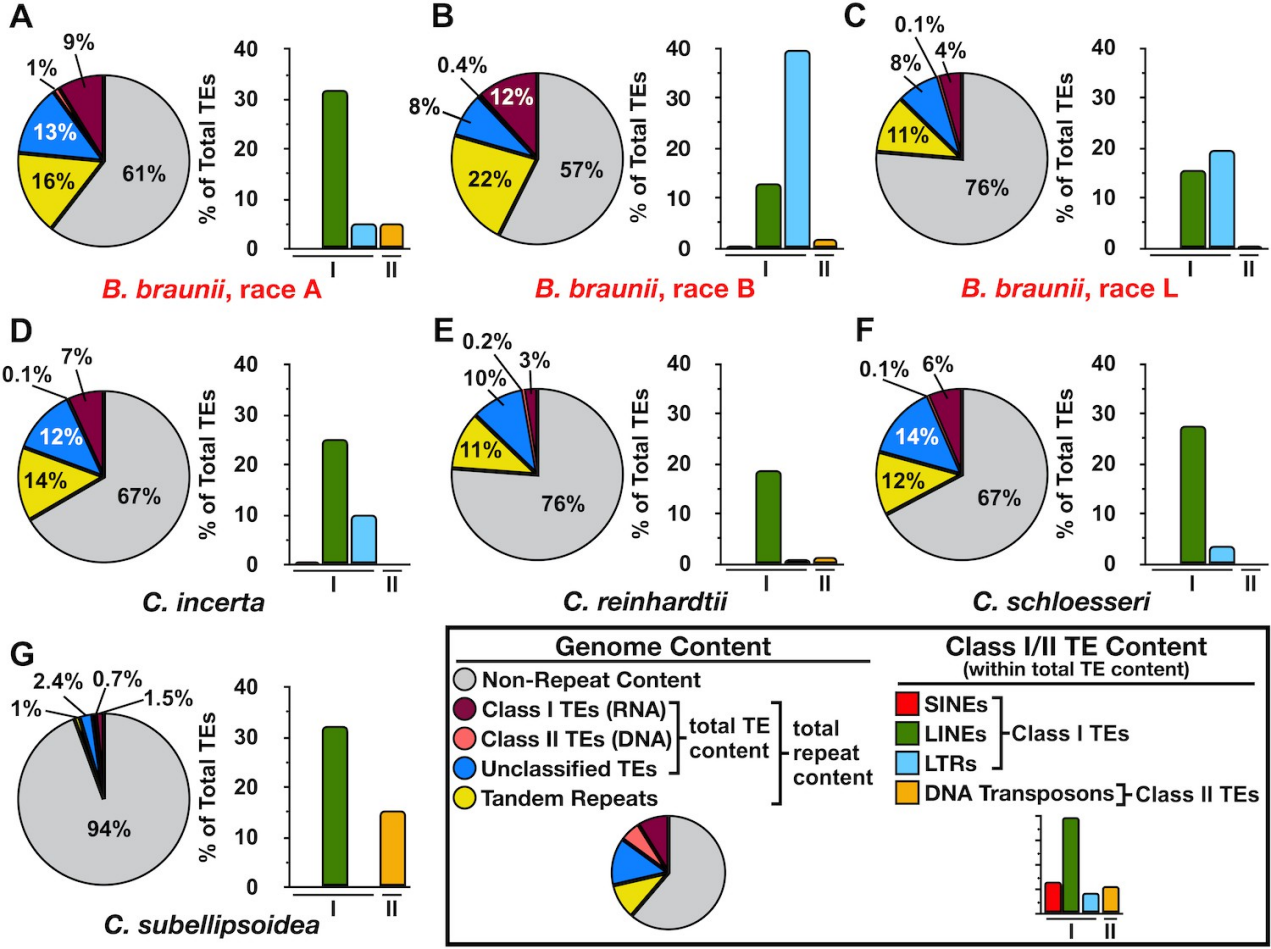
DNA repeat content of select Chlorophyta species. Total repeat content, transposable elements (TE) plus tandem repeats (TR), and the breakdown of TE type was calculated for (A) *B*. *braunii*, race A, (B) *B*. *braunii*, race B, (C) *B*. *braunii*, race L, (D) *C*. *incerta*, (E) *C*. *reinhardtii*, (F) *C*. *schloesseri*, and (G) *C*. *subellipsoidea*. The pie chart represents the amount of the genome assembly for each microalga that is made up of repeat elements by showing the total class I TEs (magenta), class II TEs (salmon), unclassified TEs (blue), total TR (yellow), and non-repeat content (grey) for each genome. The bar graph shows the breakdown of class I TEs, SINEs (red), LINEs (green), and LTRs (light blue), and class II TEs, DNA transposons (orange), as a percent of the total TE content for each race. The distribution of all repeat elements, including class I and class II TE subclasses, for each species can be found in S1 File.

The amount of DNA repeat content was examined to explain the relatively large genome sizes of the *B*. *braunii* races since a correlation between the number of putative genes and genome size was not seen (Fig 1). Transposable element (TE) and tandem repeat (TR; a.k.a. satellites and low-complexity repeats) content within the genomic space of all three *B*. *braunii* races were analyzed using RepeatModeler [46] and RepeatMasker [47] for TEs and the Tandem Repeats Finder program [48] for TRs. The B race exhibited the largest amount of total repeat content at 43% of the total genome size (Fig 3B), while the A and L races had less total repeat content at 39%, and 24%, respectively, of the total genome size (Fig 3A and 3C). The makeup of TEs in each race was also analyzed. All three races had more class I (retrotransposons) than class II (DNA transposons) TEs (Fig 3A–3C). Within the class I TEs, each race had a different major category with long interspersed nuclear elements (LINEs) dominating in the A race, long terminal repeats (LTRs) predominant in the B race, and LINEs and LTRs roughly equal in the L race (Fig 3A–3C). Short interspersed nuclear elements (SINEs) were only found in the B race as a minor component of class I TEs (Fig 3A–3C). Each race had different distributions and amounts of each subclass of class I and class II TEs (S1 File). The B race exhibited the most TR content at 22%, with the A and L chemical races at 16% and 11%, respectively (Fig 3A–3C).

To see how *B*. *braunii* total repeat content compared to other Chlorophyta species, the same DNA repeat analysis was performed on the closely related *C*. *subellipsoidea* and the three *Chlamydomonas* species. All of these species, except *C*. *subellipsoidea*, contained similar total repeat content compared to the three *B*. *braunii* races (Fig 3A–3G). While *C*. *subellipsoidea* contained little TE content, those TEs were made up of only class I LINEs and class II TEs (Fig 3G and S1 File). The three *Chlamydomonas* species contained class I LINEs as the main TE with varying amounts of LTRs and class II TEs (Fig 3D–3F). Like the observation that genome assembly size did not linearly correlate to the number genes annotated within each race of *B*. *braunii* (Fig 1), repeat content is also not linear with genome assembly size. For example, the A race has the largest genome assembly size of the three *B*. *braunii* races (Table 1), yet displayed 4% less total repeat content than the B race (Fig 3A and 3B). In contrast, *C*. *schloesseri* and *C*. *Incerta* have the largest genome size of the *Chlamydomonas* species (S1 Table in S1 File) and the most TE content (Fig 3D and 3E) analyzed here. Taken together, these data suggest TEs and TRs contribute to the observed large genome assembly sizes of the three races of *B*. *braunii*.

### Comparative genomics analysis of the *B*. *braunii* races with other algal species

#### Phylogenomic comparison of multiple chlorophyta species

Having genome assemblies of all three *B*. *braunii* races allowed for a comparative genomic analysis with other algal species. First, a phylogenetic comparison was carried out using a large dataset of genes conserved among 17 chosen algae species, including the three *B*. *braunii* races. By using a large dataset of conserved genes, more accurate phylogenetic relationships can be inferred through this type of phylogenomic analysis [49]. The species used for this analysis are listed in S1 Table in S1 File and were chosen based on having genomes, transcriptomes, and predicted gene datasets deposited and publicly available from PhycoCosm. Of the 17 species chosen, 12 were from Trebouxiophyceae (including the three *B*. *braunii* races) and five were from Chlorophyceae. To obtain the gene data set to be used in the phylogenomic analysis we utilized ChlorophytaODB v10 [50], which is a set of single-copy gene orthologs manually curated from 16 genomes of various *Chlorophyta* taxa included in the OrthoDB v10 database. At the time of this study, the ChlorophytaODB v10 had a total of 1,569 orthologous genes. A genome-wide evaluation of the 17 species revealed 1,189 of these genes were shared as single-copy orthologs while allowing any ortholog to be absent in up to three organisms.

**Fig 4:**
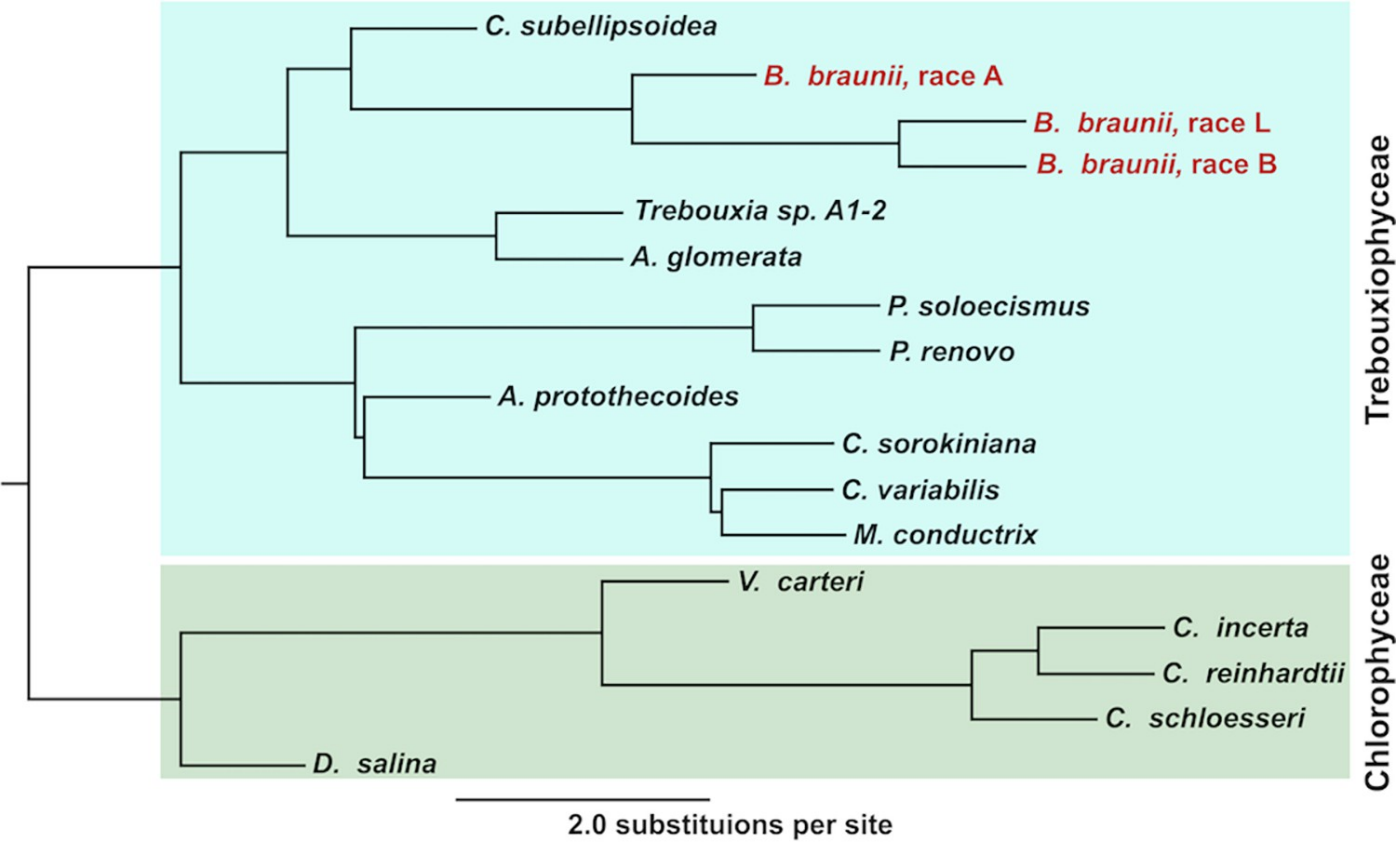
Phylogenomics of chlorophyta. Phylogenomic tree constructed using 1,189 conserved single-copy orthologs among 17 species from the Chlorophyta division. The Trebouxiophyceae class species including all three races of *B*. *braunii* are outlined in turquoise. The Chlorophyceae class species, used to root the tree, are outlined in olive green. Legend bar is in substitutions per site.

Using these conserved, single-copy orthologs, a phylogenomic tree was constructed for the 17 species analyzed (Fig 4). This tree was constructed by first generating a maximum-likelihood (ML) tree for each of the 1,189 orthologous genes. Then all 1,189 ML trees were reconciled into a single phylogenomic tree that was rooted in the Chlorophyceae sub-clade containing *Volvox carteri*, the three *Chlamydomonas* species, and *D*. *salina* (Fig 4). Phylogenetic inference placed *C*. *subellipsoidea*, as the most common species branching from the same common ancestor as *B*. *braunii* (Fig 4). The *B*. *braunii* A race was the first to diverge from a common *B*. *braunii* ancestor with the B and L races diverging from each other at a later time point (Fig 4). Additionally, the A race of *B*. *braunii* exhibits more genetic divergence from the B and L races as compared to the genetic divergence among the three species of *Chlamydomonas* (Fig 4). This phylogenomic analysis led us to conclude at this point that, at minimum, the *B*. *braunii* A race is likely a separate species from the *B*. *braunii* B and L races.

### Gene family analysis

**Fig 5:**
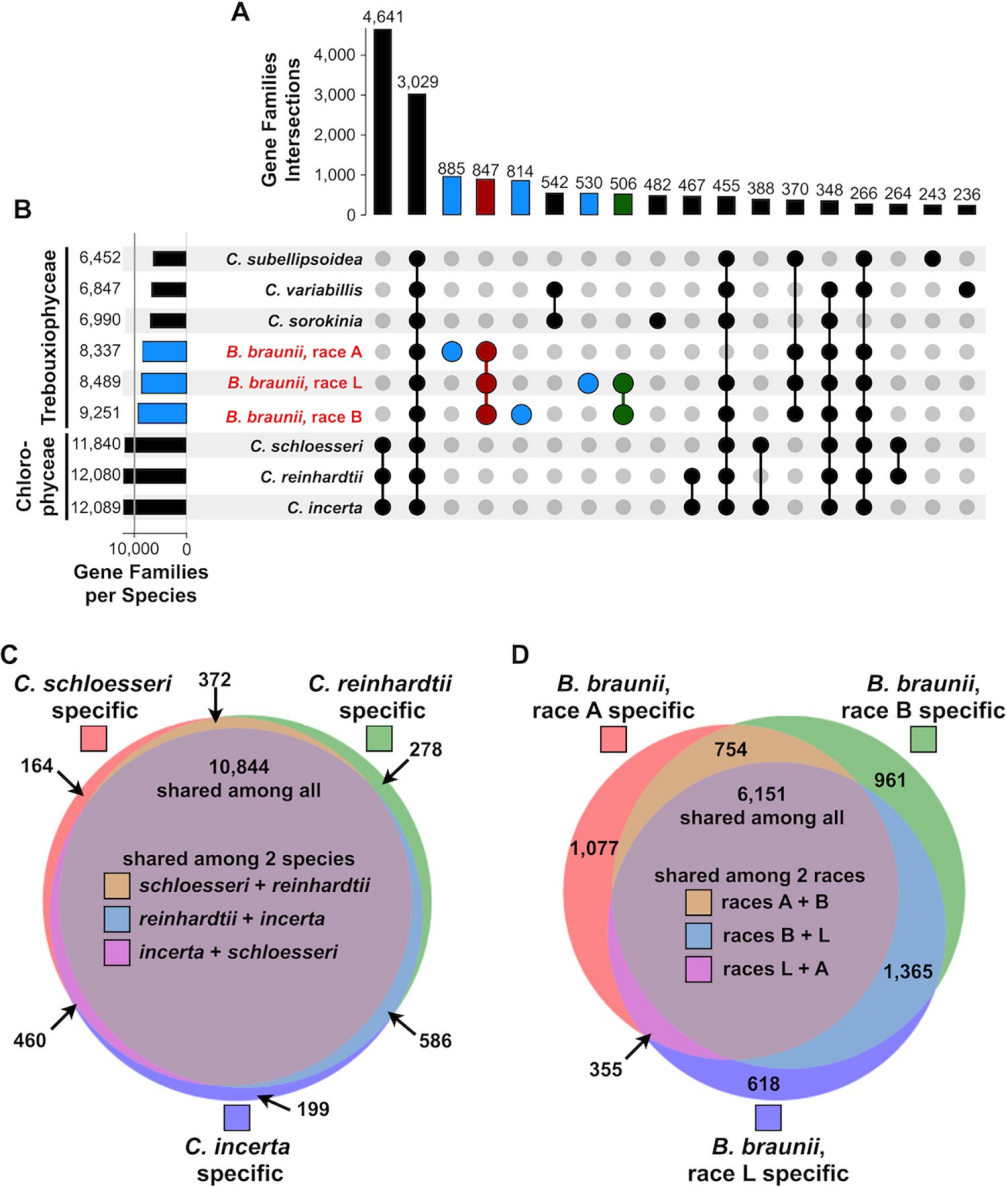
Comparative gene family analysis. UpSet plot showing the orthogroups or gene families identified by OrthoFinder grouped by species. (A) Bar chart showing the number of gene families that intersect with the given species in (B). (B) Shared gene families at different species intersections. Dots connect the species that contain the number of gene families shown in (A). Horizontal bar char in (B) corresponds to the total number of gene families with a given species. (C) and (D) Venn diagrams of shared gene families between the three *Chlamydomonas* species (C) and the three *B*. *braunii* races (D).

The number of genes and gene families shared among the three races of *B*. *braunii* in comparison to separate but closely related species can be used to support defining the A race as a separate species and provide data to determine if the B and L races are separate species. Thus, gene relationships were inferred using OrthoFinder [51], a program package that reconstructs gene orthologs and paralogs based on sequence identity shared at both the inter- and intraspecies levels into groups that are called orthogroups (hereafter referred to as gene families). This analysis was carried out on the 17 species analyzed in the phylogenomic tree and found a combined total of 226,181 genes and 22,024 gene families (S2 Table in S1 File). Next, an analysis of gene family relationships between organisms of the same genus was performed. Since the phylogenomic analysis (Fig 4) suggests the A race is likely a separate species from the B and L races, we investigated the amount of A race unique gene families not shared with the B and L races, or if the B and L races shared more gene families. An UpSet plot [52] was generated to visualize gene family intersections between 9 species: 6 Trebouxiophyceae algae: *C*. *subellipsoidea*, *C*. *variabilis*, *C*. *sorokinia*, and the three *B*. *braunii* races, and three Chlorophyceae species: the three *Chlamydomonas* species *C*. *reinhardtii*, *C*. *schloesseri*, and *C*. *incerta* (Fig 5A and 5B). The top portion of the UpSet plot (Fig 5A) groups intersections of the reconstructed gene families represented by the accompanying connected dot matrix (Fig 5B). The three *Chlamydomonas* species together had the most genus-specific gene families at 4,641, and the next highest intersection of gene families was 3,029 families shared by all 9 algae (Fig 5A and 5B). While each race of *B*. *braunii* shared 847 gene families (red dots, Fig 5B), each race had a large number of gene families specific to each race (blue dots, Fig 5B). This was not seen for the three *Chlamydomonas* species. Interestingly, the B and L races shared 506 gene families (green dots, Fig 5B), while neither the B nor L shared a large (>200) number of gene families with the A race. This supports the relationships seen between the *B*. *braunii* races in the phylogenomic analysis (Fig 4) and suggests there are more genes, and as a consequence biosynthetic/metabolic pathways, that are similar or shared between the B and L races that the A race lacks.

It would be reasonable to expect a set of gene families shared by a common ancestor of all Trebouxiophyceae and have some portion of them retained in the divergent organisms. However, there was not a large number of gene families (>200) specific only to the Trebouxiophyceae algae (Fig 5A and 5B). Interestingly, the three races of *B*. *braunii* exhibited the largest amount of genus-specific gene families of the Trebouxiophyceae algae analyzed (red dots, Fig 5B).

The large difference in unique gene families identified in each *B*. *braunii* race led us to further investigate the gene families shared between the three races in comparison to the three closely related *Chlamydomonas* species using OrthoFinder [51] and weighted Venn diagrams (Fig 5C and 5D). There were a total of 12,903 gene families identified in the three *Chlamydomonas* species, jointly they shared 10,844, or ~83%, of these gene families, and the gene families shared between any two of the *Chlamydomonas* species ranged from 372 to 586 (Fig 5C). The three races of *B*. *braunii* contained 11,281 gene families, 6,151 of which, or ~55%, were shared among the three races, and the gene families shared between any two races ranging from 355 to 1,365 (Fig 5D). Additionally, there was a greater number of gene families specific to each race of *B*. *braunii* than specific to each *Chlamydomonas* species (Fig 5C and 5D). Taken together these data indicate there is a greater degree of diversity in the gene families between the three races of *B*. *braunii* than between the three *Chlamydomonas* species. These significant differences in the genic space between the three *B*. *braunii* races offer genome-wide evidence supporting each race of *B*. *braunii* is an individual species.

#### Gene duplications

**Fig 6:**
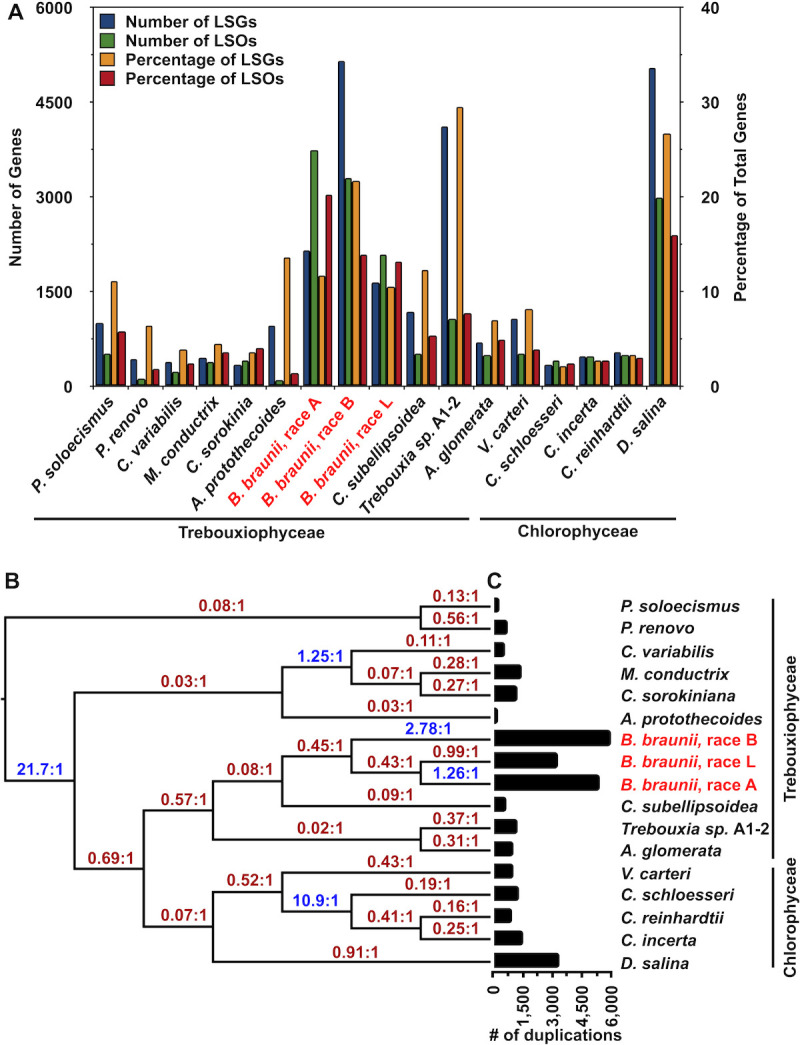
Gene duplication event analysis in chlorophyta species. (A) Distribution of the number and percentage of lineage-specific genes (LSGs) and lineage-specific orthogroups (LSOs) for each species listed. (B) Phylogenetic trees output for each single-copy ortholog from OrthoFinder were reconciled into a single tree with branch lengths mapped to gene gain/losses by Notung. At each evolutionary node break, the ratio of gene gain to loss from duplication events is labeled (e.g. 21.7:1). If the ratio indicates a net increase in gene gain it is colored in blue text. If the ratio indicates a net decrease in gene gain it is in red text. Ratios were calculated by dividing the total number of gains by losses for each branch. Tree branches represent the number of genes gained and loss from the total number of duplications calculated at each tree node. (C) Total gene duplication events per species inferred by OrthoFinder mapped as a horizontal bar chart.

Commonly, some genes cannot be placed in an orthogroup by OrthoFinder when analyzing gene families. These genes are marked as unassigned and referred to as lineage-specific genes (LSGs) unique to a given species [53]. Over time an LSG may undergo gene duplication producing lineage-specific paralogs [54, 55], and are identified by OrthoFinder as a lineage-specific orthogroup (LSO). LSG and LSO analysis was carried out on the 17 algal species used in this study. In general, the three *B*. *braunii* races have more LSGs and LSOs than the other algae analyzed except *Trebouxia sp*. A1-2 and *D*. *salina* (Fig 6A and S2 Table in S1 File). Among the *B*. *braunii* races, the B race contained the largest number of LSGs with 5,160, while the A and L races had fewer at 2,160 and 1,660, respectively (Fig 6A). For the number of genes in LSOs, the A race had the most among the *B*. *braunii* races at 3,741 with the B and L races having 3,298 and 2,095, respectively (Fig 6A). On a percentage basis, each race of *B*. *braunii* had a high percentage of LSGs and LSOs compared to most of the other algae (Fig 6A). The genes comprising the LSOs and LSGs identified in each race of *B*. *braunii* were analyzed for the presence of annotated domains using the Pfam database. Only 17.4%, 15.3%, and 18.3% of the genes in the A, B, and L races, respectively, contained an annotated Pfam domain (data not shown).

In comparison, the three closely related *Chlamydomonas* species had low and relatively equal LSGs and LSOs (Fig 6A), again indicating a large amount of genetic diversity between the three *B*. *braunii* races. Since no apparent whole genome duplication (WGD) events have been observed or inferred in *B*. *braunii* [56] it is likely the large number of LSGs and LSOs are the result of gene duplication events and subsequent functionalization [57–59]. This analysis also indicates that anywhere from 1 in 5 to 1 in 3 genes are unique to a given *B*. *braunii* race. In the *Chlamydomonas* only 1 in ~33 genes are unique to a given species between *C*. *reinhardtii*, *C*. *incerta*, and *C*. *schloesseri*. These findings further highlight the differences in the genomic space between the three *B*. *braunii* races that are not seen in the *Chlamydomonas* species.

#### Gene gain/loss analysis

To investigate if gene duplication events could be a contributing factor to the large number of unique genes identified in each race of *B*. *braunii*, the evolution of gene duplication events were tracked for all 17 algal species used in this study. 22,024 individual orthogroup phylogenetic trees from the OrthoFinder analysis were used as input to Notung [60] to determine whether a gene duplicate was retained (gain) or lost (loss). The output of Notung is a single phylogenetic tree (Fig 6B) reconciled from the OrthoFinder inputs. The total number of gains or losses for each branch in the reconciled phylogenetic tree was extracted and counted and the gain-to-loss ratios calculated (Fig 6B). Lastly, gene duplication events with high bootstrap evidence (50%) for each species were counted from the individual gene family trees from OrthoFinder (Fig 6C and S1 Fig in S1 File). *P*. *renovo* and *Picochlorum soloecismus* separated into a single clade distinct from all other species and experienced more losses than gains with a high ratio of loss to gain over any evolutionary period (Fig 6B). This may contribute to the small genome sizes of these algae compared to other *Chlorophyta* microalgae (S1 Table in S1 File). There were a few instances of net gene gain from duplications in the analysis. For example, the split delineating the other 15 species from *P*. *renovo* and *P*. *soloecismus*, the delineation of *Auxenochlorella protothecoides* from the clade containing *C*. *variabilis*, *Micractinium conductrix*, and *C*. *sorokiniana*, and the delineation of the *Chlamydomonas* species from *V*. *carteri* all had more gene gains than losses (Fig 6B and S1 Fig in S1 File). Importantly, the resulting tree topology is different between Figs 4 and 6B and arises from the different analyses that were performed. While Fig 4 is comprised of single-copy orthologs identified in at least 14 of the 17 species analyzed and infers tree topology from sequence similarity/identity, the phylogenetic inference depicted in Fig 6B focuses only on gene duplication events, and not on sequence identity itself.

The three *B*. *braunii* races experienced varying levels of gene gain/loss from duplication events. The B race experienced the highest gain-to-loss ratio of 2.78 gains per loss from duplication events, the A race experienced 1.26 gains per loss, and the L race was the only race to experience a higher ratio of loss to gain from gene duplication events, at 0.99 gains per loss (Fig 6B). In terms of absolute numbers of gene duplications, *B*. *braunii* experienced the largest amount of gene duplication events among the 17 species analyzed (Fig 6C and S1 Fig in S1 File). Specifically, the B race had the largest amount of gene duplication events, followed by the A race, while the L race had the fourth-largest number of genome duplication events falling below *D*. *salina* (Fig 6C and S1 Fig in S1 File). This is interesting considering the *D*. *salina* genome size is nearly triple that of the largest *B*. *braunii* A race genome assembly (S1 Table in S1 File). A large amount of *B*. *braunii* gene duplication events (Fig 6C and S1 Fig in S1 File) may have given rise to their significantly larger genomes when compared to other Trebouxiophyceae algae (S1 Table in S1 File). Additionally, the varying ratios of gain to loss from gene duplication events between the three races suggest that over an evolutionary period, the races of *B*. *braunii* have experienced significantly different changes in their genic space. Most *Chlorophyta* species, such as the closely related *Chlamydomonas* species, appear to have lower levels of gene duplication events, and never experienced retention of genes through duplication events at the time of species delineation [61]. The finding that the A and B races of *B*. *braunii* had more retention of genes over losses from duplication events is the first report of this finding for a C*hlorophyta* species. Since the B and L races have different gene gain-to-loss ratios (Fig 6B), it suggests the B and L races underwent divergent genome expansion or reduction events after delineation from a common ancestor. These differences mark another separation between the B and L races.

### Analysis of hydrocarbon biosynthesis gene distribution within the three races of *B*. *braunii*

Historically, it has been noted that the three races of *B*. *braunii* differ from each other mainly by the type of hydrocarbon produced by each race [7, 8, 62]. That is to say, the hydrocarbons of one race are not found in the other two races. It has been presumed this is due to each race having only the genes specific to the production of the hydrocarbons in that race. However, it is possible that hydrocarbon biosynthesis genes from one race exist in the other races but are not expressed. With the genome sequence for each race, the existence of these genes in each race of *B*. *braunii* can be analyzed. Thus, each *B*. *braunii* race was analyzed for the presence of known hydrocarbon biosynthesis genes from each race using HMMER searches with specific Pfam protein domains and BLAST searches for hydrocarbon genes from each race.

#### Distribution of potential A race hydrocarbon biosynthesis genes within *B*. *braunii*: Long-chain fatty acid synthesis genes

Though the hydrocarbon biosynthesis genes responsible for alkadiene/triene biosynthesis in the A race of *B*. *braunii* have yet to be identified, evidence suggests that these hydrocarbons are derived from the elongation of fatty acids to produce very long chain fatty acids (VLCFA) followed by cleavage of the carboxylic acid moiety to generate an alkene with a terminal double bond [10, 11, 13]. Production of VLCFAs through FA elongation has been well studied in many organisms, is highly conserved, the genes/enzymes involved have been identified, and is a four-step process [63]. The first enzyme, 3-ketoacyl-CoA synthase (KCS) adds an acetate moiety from malonyl-CoA to the FA generating a β-carbonyl, the second enzyme, 3-ketoacyl-CoA reductase (KCR), reduces the β-carbonyl to a β-hydroxyl, the third enzyme, 3-hydroxyacyl-CoA dehydratase (HCD), dehydrates the β-hydroxyl to a C=C bond, and the fourth enzyme, *trans*-2,3-enoyl-CoA reductase (ECR), reduces the C=C bond [63]. These 4 reactions repeat until the needed acyl chain length is produced. The KCR, HCD, and ECR genes are highly conserved, are single-copy genes, and accept a broad range of acyl chain lengths [63]. The KCS genes belong to a large gene family [64, 65] and each encoded enzyme can be very selective for substrate acyl chain lengths [66]. KCS genes can be subdivided into two subfamilies: the plant specific fatty acid elongase (FAE)-type and the more universal elongation deficient (ELO)-type [63, 67, 68].

As a starting point and in an attempt to identify A race genes/enzymes specific for alkadiene/triene production we analyzed each race for the presence of FAE- and ELO-type KCS proteins, and the single-copy KCR, HCD, and ECR proteins. The Pfam ketoacyl-synt domain (Pfam: PF08392) was used to search for the FAE-type KCS sequences and a total of 63 targets distributed among the three *B*. *braunii* races were found with 16, 20, and 25 for the A, B, and L races, respectively (S2 Fig in S1 File). A search for ELO sequences in each race using the Pfam ELO domain (Pfam: PF01151) revealed a total of four targets in the A race, eight in the B race, and three in the L race (S3 Fig in S1 File). The KCR and HCD genes were identified by BLAST using the proteins for single-copy genes identified in *Arabidopsis thaliana* (NP_564905.1, and NP_193180.1 respectively) that were downloaded from NCBI and used as queries in a BLAST+ protein alignment. For ECR, the Pfam enoyl-CoA reductase domain (Pfam: PF12241) was used to search for homologous sequences in each race. These searches yielded more than one target sequence per race, which were narrowed down to a single protein sequence when possible based on nearest homolog analysis against the NCBI non-redundant protein database. The KCR protein search revealed a single target in each race. However, for the HCD and ECR searches a single target could be identified in only two of the three races. For the HCD search, a single target was found in the A and L races, but not in the B race. For the ECR search, a single target was found in the A and B races, but not in the L race. From this analysis, it is difficult to determine if any of these sequences are specific for the A race and contribute to alkene biosynthesis. A detailed functional analysis of the encoded genes will be required in the future.

#### Distribution of B and L race hydrocarbon biosynthesis genes within *B*. *braunii*: Squalene synthase/phytoene synthase family genes

Squalene synthase (SS) condenses two molecules of C_15_ farnesyl diphosphate (FPP) to produce C_30_ squalene as the first committed step in sterol biosynthesis in eukaryotic organisms and is generally found as a single-copy gene in yeast and humans [69, 70], but multiple copies have been isolated in some plant species [71–75]. Phytoene synthase (PYS) catalyzes a reaction similar to SS by utilizing C_20_ geranylgeranyl diphosphate (GGPP) to produce C_40_ phytoene [76], which is structurally similar to squalene. Thus, PYS can be considered an SS-like sequence, and most photosynthetic organisms have a single-copy of *PYS*. Each race of *B*. *braunii* has been shown to contain a single SS gene [18, 77]. In addition to squalene production by the conventional SS, biosynthesis of the triterpene hydrocarbon botryococcene in the B race has been shown to use the SS-like genes SSL-1 and SSL-3 [78], In relation to this, there is an alternative route for squalene production by the combination of SSL-1 and SSL-2 in the B race [78]. Another SS-like enzyme, lycopaoctaene synthase (LOS), is responsible for lycopadiene production in the L race [18]. *PYS* genes have not yet been published for any race of *B*. *braunii*. All three races were screened for the presence of all SS, SS-like, and PYS protein sequences using the curated SQS-PSY Pfam domain (Pfam: PF00494). The analysis found a single SS sequence for each race, sequences corresponding to the SSL1, 2, and 3 were only found in the B race, and LOS was only found in the L race (S4 Fig in S1 File). Thus, the hydrocarbon biosynthesis enzymes for the B and L races are only found in those races. Additionally, a single PYS was found in each race (S4 Fig in S1 File).

#### Meiosis genes present in the genome assemblies of *B*. *braunii*

To date, no evidence has been presented indicating that any race of *B*. *braunii* displays sexual reproduction behavior [20]. Within Chlorophyta, there are several species that are suspected to display cryptic sex [79]. For example, there are several Trebouxiophyceae species that encode meiosis genes within their genomes including several analyzed in this study: *A*. *protothecoides*, *C*. *variabilis*, and *C*. *subellipsoidea* [79]. To investigate if *B*. *braunii* contains the ability for sexual reproduction, the genome assemblies of each race of *B*. *braunii* were searched for nine essential meiotic genes: DMC1, HOP1, HOP2, MER3, MND1, MSH4, MSH5, REC8, and SPO11 [79]. Orthologs for each of these genes, with supporting Pfam domains, were identified in the three races of *B*. *braunii*, suggesting the presence of a meiosis pathway in the genomes of all three races and cryptic sex in this alga.

## Discussion

The current understanding of *B*. *braunii* has been largely limited to studying morphological and hydrocarbon content/biosynthesis differences between the three races. This limitation is due to a lack of “omics” datasets for each race, and recently only the B race of *B*. *braunii* had a sequenced genome available [34]. The current study aimed to obtain genomic sequences for the A and L races to perform a comparative genomic analysis of the three *B*. *braunii* races. This allowed us to probe for differences between the races other than hydrocarbon biosynthesis. Comparative genomics has long been leveraged to investigate relationships between similar organisms. *Arabidopsis thaliana* is a single species made up of many ecotypes (natural variants), classified based on geographical and subtle phenotypic differences [80]. These ecotypes have been investigated at the genomic sequence and TE level [81–83]. Multiple ecotypes of *A*. *thaliana* had near identical distributions and composition of TEs in all five chromosomes [83], yet the only large difference at the genomic level is a moderate variance in polymorphisms between genomes [81]. One major difference observed among the ecotypes has been a large variation in transcriptional response to environmental stressors, likely a result of different selective pressures from their respective geographical environments [84]. *A*. *thaliana* ecotypes are somewhat akin to the *B*. *braunii* chemical races; the races have multiple strains isolated from different waters [20]. However, unlike the *A*. *thaliana* ecotypes, our comparative genomic analysis revealed substantial differences between the three races in their genomic space indicating the races are separate species.

Our findings suggest the three races divergently evolved from a common ancestor and each race contains substantial variability within the genomic space for each to be considered a separate species (Figs 1, 3A-3C and 6A). By comparison, the three *Chlamydomonas* species included in our analyses were more similar to each other in the genomic space than the three *B*. *braunii* races are to each other (Fig 5C and 5D). Thus, we suggest a reclassification of the *B*. *braunii* races into three distinct species of the genus *Botryococcus*.

### New *Botryococcus* species names

The *B*. *braunii* species was first named in 1849 by Friedrich Traugott Kützing [85] and was named after Alexander Karl Heinrich Braun, a 19^th^ century botanist [86]. The A and B races of *B*. *braunii* were classified in 1985 [62] and the L race in 1987 [8] based on the type of hydrocarbons produced in each race. Based on the data presented here we created new species names for the three races of *B*. *braunii* using the general rule for generating species names that the species name should match the gender of the genus name [87]. *Botryococcus* is considered as male [86]. Thus, we propose the following species names. The A race is renamed *Botryococcus alkenealis*. The general term for the A race hydrocarbons, alkene, is used with the Latin suffix -*alis* added, which is male or female and means pertaining to, i.e. pertaining to alkenes. The B race retains the name *Botryococcus braunii* to preserve the history of being the first to have its hydrocarbons identified, i.e. botryococcenes [88]. The L race is renamed *Botryococcus lycopadienor*. The root of the hydrocarbon name for the L race, lycopadiene, is used with the Latin suffix *-or*, which is a male suffix with no descriptive meaning. Hereafter, the three races of *B*. *braunii* will be referred to by these new species names and all researchers in the *Botryococcus* field are encouraged to adopt this new nomenclature.

### Evidence in support of the chemical races as three different species

#### Genome repeat content

It is well established that repeat content is the largest driving force in genome size and is responsible for some of the largest observed plant genomes to date [89, 90]. In the core-*Chlamydomonas* species, extensive TE curation revealed large differences in TE content among closely related species relative to *C*. *reinhardtii* [91]. We observed similar differences between the three *Botryococcus* species (Fig 3A–3C and S1 File), and the large repeat content of the three *Botryococcus* species likely contributes to their large genome sizes. Additionally, the large gene count annotated in the *B*. *braunii* genome likely contributes to its large assembly size. Future studies on the repeat content in *Botryococcus* species should look at multiple representative strains from each species (i.e. each former race) to investigate whether strains of the same species contain similar or varying amounts of repeat content. Variation of TE content is observed in other plant species of the same genus, like those within the genuses of *Oryza*, *Zea*, and *Arabidopsis* [92–94]. The large variation of TE content between *B*. *alkenealis*, *B*. *braunii*, and *B*. *lycopadienor* supports our theory that the three former chemical races are three distinct species. While automated TE annotation is convenient and fast, it can be error prone and cannot replace manual TE curation. Thus, our TE analysis likely represents only a portion of the actual TE content present in the *Botryococcus* genomes. Manual curation of TEs would generate a more robust cataloging of the TE content in each *Botryococcus* species and provide a better picture of the contribution of repeat content to *Botryococcus* genome size.

#### Genome-wide phylogenomics

Prior 18S rRNA-based phylogenetic inferences have suggested the three races are three different species [9, 16, 17, 20, 95]. These studies showed that *B*. *alkenealis* (A race) diverged earlier and separated from a common ancestor with *B*. *braunii* and *B*. *lycopadienor* (B and L races, respectively). Nearly all these studies concluded that *B*. *alkenealis* was likely a different species but there was no observable separation between the *B*. *braunii* and *B*. *lycopadienor*. However, these observations are limited since the phylogenetic trees generated were based on a single gene limiting the ability to infer divergent evolution [49]. In our phylogenomic analysis (Fig 4), the use of 1,189 highly conserved orthologs indicates a clear separation between the three *Botryococcus* species and supports the theory that *B*. *braunii* and *B*. *lycopadienor* diverged later than *B*. *alkenealis* from a common ancestor. To date, this is the first genome-wide evidence supporting the theory of divergent evolution between the *Botryococcus* species (i.e. the three races). This is further supported by the divergence of specialized secondary metabolite biosynthetic pathways related to hydrocarbon biosynthesis. An analysis using derivatives of the *B*. *braunii* and *B*. *lycopadienor* hydrocarbons as biomarkers in sediments concluded that the genes for these hydrocarbons likely evolved no more than 55 million years ago [96]. This analysis also shows that *B*. *braunii* and *B*. *lycopadienor* diverged separately but at a similar rate from a later shared ancestor that appeared after *B*. *alkenealis* diverged. It is important to note that even though the hydrocarbons biosynthesized by *B*. *braunii* and *B*. *lycopadienor* are different they are both derived from isoprenoid biosynthetic pathways and utilize similar enzymes that may have arose from gene duplication and neo-functionalization [18, 78]; the SS-like SSL and LOS genes in *B*. *braunii* and *B*. *lycopadienor*, respectively. This close similarity in the biosynthesis of their major metabolite reflects their divergence with each other from *B*. *alkenealis* (Fig 4). Generating chromosome resolution genome assemblies for all three *Botryococcus* species could further isolate similarities and differences between *B*. *braunii* and *B*. *lycopadienor*.

#### Gene content

We add evidence for separate species based on the comparison of gene content between the *Botryococcus* species. The genome-wide gene family comparison of the 17 Chlorophyta species (Figs 5 and 6) revealed large differences in gene family content between the three *Botryococcus* species that are larger than the differences between related taxa that are classified as different species. For example, the three *Chlamydomonas* species share ~83% of their total gene families, while all three *Botryococcus* species share ~55% of total gene families (Fig 5C and 5D). This data combined with previous genetic divergence rates of 18S rRNA genes [9] supports our proposal of three separate *Botryococcus* species.

All three *Botryococcus* species had a large number of gene duplication events leading to the acquisition of new genes (Fig 6C). However, *B*. *alkenealis* and *B*. *braunii* had approximately 2-fold more gene duplication events than *B*. *lycopadienor*, and all three *Botryococcus* species had nearly 24-36% of their total gene pool classified as specific to that species (Fig 6A). Additionally, the three *Botryococcus* species underwent different rates of gene duplication/loss ratios over a recent evolutionary period (Fig 6B). Taken together these observations suggest that after duplication of orthologs and paralogs these genes underwent a high level of neo-functionalization and/or sub-functionalization in the *Botryococcus* genus, giving rise to a large number of species-specific gene families and genes [53, 57, 58]. The three *Chlamydomonas* species analyzed here had similar levels of gene duplication events and nearly identical gene gain/loss ratios at the point of delineation (Fig 6B and 6C). In addition, the three *Chlamydomonas* species all had lineage-specific genes, however, the total amount was <10% of the total gene pool (Fig 6A and 6B). The three *Botryococcus* species all had relatively large levels of lineage-specific genes identified (Fig 6A and 6B). The role these lineage-specific genes/gene families and their expansion play in eukaryotic organism evolution has been well established [97]. The vast amount of lineage-specific genes/gene families among the *Botryococcus* species has likely played a large role in the differences we have observed in the genomic space and supports our proposal of separate *Botryococcus* species.

It has been previously established that TE content and whole genome duplication (WGD) events are linked to the evolvability and complexity of an organism at the genome level in both maize and rice [98, 99]. While no WGDs have been observed, nor is the chromosome numbers known, for any of the three *Botryococcus* species, it is too early to speculate about the level of genome duplication that may have occurred in *Botryococcus*. Our data presented here including the phylogenomic placement, genome sizes, gene duplication events, and TE content of the three *Botryococcus* species suggests that some large level of genomic sequence duplication has occurred. However, this is speculatory and requires further investigation of the *Botryococcus* genomes including obtaining chromosome resolution assemblies and karyotyping each *Botryococcus* species.

## Future directions

Additional genomic analyses within the *Botryococcus* genus are required to resolve several issues related to species and races. For example, phylogenetic analysis of the previously mentioned S race [9] using the 18S rRNA gene [9, 20] shows the S race is closely related to and may be a member of *B*. *lycopadienor* (i.e. the L race). A study published in 1992 suggested the existence of several different species of *Botryococcus* based solely on morphological differences in cell and colony shape and size [100]. However, since *Botryococcus* colony and cell morphology can be easily influenced by environmental factors causing observable differences within the same race/strain [101], these proposed species may not actually be separate species. For example, one of the proposed species, *B*. *terribilis* [100, 102] may actually be a strain of *B*. *braunii* (i.e. the B race) based on subsequent studies analyzing hydrocarbon content and phylogenetic placement [103, 104]. Additionally, phylogenetic studies [9, 20] have shown that *B*. *braunii* can be divided into two distinct subclades, suggesting *B*. *braunii* may consist of two separate species. Genomic sequencing, assembly, and comparative analysis on the S race, *B*. *terribilis*, members of each *B*. *braunii* subclade, and other proposed *Botryococcus* species is needed to resolve their positions within the *Botryococcus* genus.

## Materials and methods

### Culturing of *B*. *braunii*

Culture conditions for *B*. *alkenealis*, *B*. *braunii*, and *B*. *lycopadienor* were followed as described previously [18]. Briefly, *B*. *alkenealis*, (race A, Yamanaka strain [105]), *B*. *braunii* (race B, Showa strain [106]), and *B*. *lycopadienor (*race L, Songkla Nakarin strain [8]) were grown in modified Chu 13 medium under continuous aeration with filter sterilized air mixed at 2.5% CO_2_. The composition of the modified Chu 13 medium was as follows: KNO_3_ (0.4 g/L), MgSO_4_ · 7H_2_O (0.1 g/L), K_2_HPO_4_ (0.052 g/L), CaCl_2_ · 2H_2_O (0.054 g/L), FeNa EDTA (0.01 g/L), H_3_BO_4_ (2.86 mg/L), MnSO_4_ · H_2_O (1.54 mg/L), ZnSO_4_ · 7H_2_O (0.22 mg/L), CuSO_4_ · 5H_2_O (0.08 mg/L), NaMoO_4_ · 2H_2_O (0.06 mg/L), CoSO_4_ · 7H_2_O (0.09 mg/L). Cultures were grown under a light:dark cycle of 12:12h with a constant light intensity of 120 μE m^-2^s^-1^. Alga cells were subcultured by inoculation of 100 mL from a 6-week-old culture into 750mL of fresh medium.

### DNA isolation and sequencing

Algal cells were harvested by vacuum filtration through a 5-μm or 10-μm nylon microsieve (BioDesign Inc. of New York) for *B*. *lycopadienor* and *B*. alkenealis/*B*. *braunii*, respectively. Cells were either used directly or snap-frozen in liquid nitrogen and stored at -80°C until further use. High molecular weight (HMW) genomic DNA (gDNA) free of RNA and polysaccharides, was isolated from harvested biomass using a protocol we developed specifically for *Botryococcus* species [35] that was adapted from previous studies [107, 108]. Briefly, harvested algae biomass was macerated by mortar and pestle in the presence of liquid nitrogen to a fine powder. Then 100-110mg of macerated biomass was resuspended in 1 mL of sorbitol wash buffer (100 mM Tris-HCl pH 8.0, 0.35 M sorbitol, 5 mM EDTA pH 8.0, 1% (w/v) polyvinylpyrrolidone (PVP-40)), sonicated at 30% power for 25 seconds (strong enough to homogenize the sample, but not to lyse the cells), centrifuged at 2,500 x *g* for five minutes at room temperature and the supernatant discarded. Due to the hydrocarbons in the sample, the pellet floats on top of the supernatant. Care was taken to remove the supernatant without disturbing the floating pellet. The washed pellet was then resuspended by vortexing for 5 seconds in 700 μL of pre-warmed (65°C), extraction buffer (100 mM Tris-HCl pH 8.0, 3 M NaCl, 3% CTAB-cetyl trimethylammonium bromide, 20 mM EDTA, 1% (w/v) Polyvinylpyrrolidone). Resuspended pellets were then incubated for 30 minutes in a 65°C water bath, mixed by inversion every 10 minutes, and allowed to cool to room temperature for 5 minutes. Then 700 μL of 24:1 (v:v) chloroform: isoamyl alcohol (CIA) was added, mixed by vortexing for 10 seconds, and then centrifuged at 2,500 x *g* for 10 minutes at room temperature. The aqueous phase was removed and treated with Rnase A (0.1 mg/mL) at 37°C for 15 minutes with mixing by inversion every 5 minutes, 500 μL of CIA added with mixing by inversion, centrifuged at 13,000 x *g* for 10 minutes at 4°C, and the aqueous phase was recovered and transferred to a fresh tube. HMW gDNA was then precipitated by the addition of 1/10 volume of 3M sodium acetate (pH 5.2), and 2/3 volumes of cold (-20°C) isopropanol, the sample was mixed by inverting 10 times, and incubated overnight at -20°C. The samples were then centrifuged at 13,000 x *g* for 10 minutes at room temperature, the supernatant discarded, the pellet air-dried, and the pellets washed with 1 mL of 70% ethanol followed by centrifugation at 13,000 x *g* for 10 minutes at 4°C. The HMW gDNA pellet was then dried under vacuum for 10 minutes, resuspended in 100 μL of nuclease-free water, and either stored at -80°C or prepped for sequencing.

Library preparation and sequencing for Illumina-based sequencing was performed on the Illumina HiSeq2000 platform by the Texas A&M AgriLife Genomics and Bioinformatics Service (TxGen). Oxford Nanopore-based sequencing was performed by the Sequencing Technologies and Analysis core facility at the Cold Spring Harbor Laboratory. For this sequencing, DNA concentration was determined via fluorometry and small/fragmented gDNA (<25kb) was removed using the Circulomics short read eliminator kit (SS-100-101-01). Samples were barcoded and adaptors ligated with the native barcode kit (Oxford Nanopore Technologies, EXP-NBD104). Sequencing was performed on the Oxford Nanopore PromethION platform, using R9.4.1 flowcell biochemistry and base calling was done with ONT’s proprietary Guppy (v4). In total, two flow cells were run due to complications with the quality of sequencing data on the first flow cell, the lower quality flow cell run will be referred to as flow cell 1 and the higher quality flow cell referred to as flow cell 2. Both flow cells’ data were used in the final genome assemblies for *B*. *alkenealis* and *B*. *lycopadienor* given recovery of read quality after read trimming. Both sets of reads from each flow cell were subjected to the same pre-assembly trimming process, however, flow cell 1 had sufficiently fewer reads post-trim than flow cell 2 reflecting the overall lower quality of the reads obtained.

### *De novo* genome assembly for *B*. *lycopadienor* (L race)

The raw fastq files from the Nanopore sequences were trimmed with Porechop (v0.2.4) to remove any remaining non-gDNA sequences and poor-quality bases. The L race genome assembly was performed using the Flye long-read assembler [109] with the trimmed long-read sequences as input. The resulting scaffolds and contigs were then polished with the short-read Illumina DNA sequencing data that was trimmed for barcode, adapter, and poor quality sequence using Trimmomatic [110]. Scaffold polishing was done iteratively twice with Pilon [111], with the output of the first round of polishing used as the input for the next round of polishing. Scaffolds/contigs belonging to symbiotic or organelle DNA [112] were identified and removed using Blobtools (v1.1) [113]. Briefly, scaffolds/contigs that displayed GC% and k-mer frequency and were annotated taxonomically as non-green microalgae were individually reviewed for confirmation before removal. The nuclear draft genome was then masked using a combination of RepeatModeler (v1.0) [46] and RepeatMasker (v4.0) [47]. First, a *de novo* repeat library was assembled using RepeatModeler. The repeat library was then used to mask the genome with RepeatMasker using *B*. *braunii* B race CDS [34] (JGI: Bbraunii_502_v2.1.cds.fa) as trusted sequences to prevent over-masking by RepeatMasker. Finally, assembly metrics and quality were assessed using Quast [114] and BUSCO v5 [115], respectively. ChlorophytaODBv10 [50] was used as the ortholog database for BUSCO analysis.

### Hybrid *de novo* genome assembly for *B*. *alkenealis* (A race)

The genome assembly pipeline used for *B*. *lycopadienor* was applied to *B*. *alkenealis* but did not result in a high-quality assembly. Thus, a modified approach was taken to produce a higher quality assembly for *B*. *alkenealis* to maximize the final N50, minimize the final L50, and reduce the number of “N”’s in the unmasked assembly. Trimming of both the Nanopore and Illumina sequencing data along with contig/scaffold assembly of the long read sequences by Flye remained the same as was done for *B*. *lycopadienor*. The hybrid assembler MaSuRCA (v4.0.1) [116] was used to generate a hybrid genome assembly from both the long and short read sequences as input. Then both the Flye assembly and MaSuRCA assembly were merged and de-replicated using Quickmerge [117]. The resulting merged assembly was then polished twice iteratively with the short-read sequence using Pilon. Removal of non-nuclear DNA, masking, and quality/metric assessment was done as described for the *B*. *lycopadienor*.

### Attempts at genome assembly for the *B*. *braunii* (B race)

Attempts were made to improve the current *B*. *braunii* (B race) genome assembly [34]. The newly generated Nanopore sequencing data were used in identical workflows that were used for *B*. *lycopadienor* and *B*. *alkenealis* genome assemblies mentioned above. Unfortunately, neither resulted in an improved assembly. While N50 values were slightly higher than the current metrics, both contig number and L50 values were larger than their respective values in the current *B*. *braunii* (B race) genome assembly. BUSCO analysis also showed a higher amount of fragmented orthologs than the current assembly. An additional 10 assembly approaches were tested including a reference-guided genome assembly approach [118] using the current assembly as “trusted” contigs for inputs into MaSuRCA, Flye, and Canu assemblers. None of these advanced assembly tactics yielded an improved genome assembly for *B*. *braunii*. Likely, the Nanopore sequencing data did not contain enough sequencing depth to resolve the complexities of the *B*. *braunii* genome [34]. Future attempts at genome sequencing and assembly into *B*. *braunii* will need more long-read sequencing data and optical mapping to resolve complex, repeat-rich portions of the genome.

### RNA isolation and sequencing

*B*. *alkenealis* and *B*. *lycopadienor* cultures were grown and biomass was harvested as described above. Total RNA was isolated and purified using the TRIzol Reagent (Thermofisher). The resulting RNA pellet was washed with 75% ethanol and then dried using a speed vac. To remove the polysaccharides from the samples, the RNA pellet was resuspended in 2M LiCl and centrifuged at 10,000 x *g* at room temperature for 10 minutes. The supernatant containing polysaccharides was discarded and the pellet retained. This process was repeated until the RNA pellet size remained the same after each wash step. The washed pellet was then resuspended in 1x TE buffer and extracted against and an equal volume of 1:1:1 phenol/chloroform/isoamyl alcohol. The suspension was then centrifuged at 10,000 x *g* at room temperature for 10 minutes with the pellet discarded and the supernatant saved. A final extraction against an equal volume of chloroform was done followed by centrifugation at 10,000 x *g* and the supernatant saved. Finally, the RNA was precipitated with 0.1 volumes of 3M sodium acetate and 2.5 volumes of 100% ethanol. The solution was centrifuged at 10,000 x *g* for 10 minutes at room temperature and the pellet was saved. The RNA pellet was resuspended in Rnase-free water and treated with Dnase (1 unit/μg RNA), incubated at 30°C for 30 min, treated with 1:1:1 phenol/chloroform/isoamyl alcohol, precipitated with sodium acetate and ethanol as described earlier, the DNA-free final RNA pellet was resuspended in nuclease-free water, and stored at -80°C until further use. RNA pellets were sent for sequencing through the National Alliance for Advanced Biofuels and Bioproducts (NAABB) to the Los Alamos National Laboratory for sequencing on the Illumina platform [119, 120].

### Genome-guided transcript assembly for *B*. *alkenealis* and *B*. *lycopadienor*

RNA-seq data were trimmed of adapters and low-quality bases using Trimmomatic. Trimmed reads were then mapped to the corresponding genome assemblies using HISAT2 [121] with default settings. The resulting BAM file was indexed and fed as input for use in the Trinity suite [122] run in genome-guided mode. The Trinity raw assembled transcripts were then filtered for low-expression isoforms using Trinity’s built-in scripts, and the removal of redundant transcripts was done with CD-HIT-EST [123]. Final draft transcriptomes were assessed for quality and metrics by Quast and BUSCO, respectively, run in transcript mode. ChlorophytaODBv10 was used as the ortholog database for BUSCO analysis.

### Annotation of genome features

Genes were predicted and annotated using the BRAKER2 pipeline [124–133]. Briefly, BRAKER2 was run in “evidence-based” mode where RNA-seq data was first mapped to a hard-masked version of the genome assemblies using HISAT2 [121]. The resulting BAM file was used as input into BRAKER2 with default parameters and the output was saved in GTF format for all three Botryococcus species. BRAKER2 was also used for the untranslated region (UTR) analysis using the GUSHR module. At the time of analysis, BRAKER2 did not support automatic intronic region annotation. So, intronic sequences were detected and annotated using a custom python script with the BRAKER2 GTF output files. Due to BRKAER2’s increased capability of fully automatic training of the gene prediction toolsets and leveraging RNA-seq and protein homology information into the final gene prediction sets, the decision was made to re-annotate the *B*. *braunii* (B race) v2 genome assembly, using this increased capability that was not available at the time of the original release of the v2 assembly. This re-annotation gave a different final number of genes when compared to the v2 genome assembly files. This new annotation predicted 23,685 genes while the v2 genome annotation predicted 20,765 genes as seen in Fig 1. Transposable element content was annotated using RepeatModeler [46], and RepeatMasker [47]. DNA tandem repeats (satellites) were annotated by Tandem Repeats Finder [48] using default parameters. In all three *Botryococcus* species, genes were scanned for coding sequence overlaps with TEs at an overlap threshold of ≥70%. This was achieved by converting the RepeatMasker output file and BRAKER2 GFF/GTF output files to the BED format. BEDTools [134] was then used to identify overlapping regions using the “intersect” program with the “-f 0.70” flag.

### Phylogenomic analysis

The approach for a mass phylogenomic analysis using multiple microalgae species was modified slightly from a previous study [91]. Briefly, protein sequence files for each species analyzed in this study (*B*. *alkenealis*, *B*. *braunii*, *B*. *lycapdienor*, *Asterchloris glomerata*, *Auxenochlorella protothecoides*, *Chlamydomonas incerta*, *Chlamydomonas reinhardtii*, *Chlamydomonas schloesseri*, *Chlorella sorokiniana*, *Chlorella variabilis*, *Coccomyxa subellipsoidea*, *Dunaliella salina*, *Micractinium conductrix*, *Picochlorum renovo*, *Picochlorum soloecismus*, *Trebouxia sp*. *A1-2*, *Volvox carteri*) were screened by BUSCO in protein mode using the ChlorophytaODBv10 database [50]. Single-copy orthologous proteins that were conserved in at least 13 of the 17 species were extracted, counted, and analyzed. Multiple sequence alignments (MSAs) of each ortholog set were produced using MAFFT v7 [135]. Maximum likelihood trees were inferred for each ortholog set from the corresponding MSAs using IQTREE [136] run with the following parameters: `-m MFP -bb 1000 -T 5`. The final set of 1,189 ortholog trees was reconciled into a single alternative species tree using ASTRAL-III [137]. The final phylogenomic tree was rooted in the Chlorophyceae sub-clad*e*.

### Comparative genomic analysis

Protein sequence files for *B*. *alkenealis*, *B*. *braunii*, and *B*. *lycopadienor* were generated using BRAKER2 as described above. The remaining 14 species (*A*. *glomerata*, *A*. *protothecoides*, *C*. *incerta*, *C*. *reinhardtii*, *C*. *schloesseri*, *C*. *sorokiniana*, *C*. *variabilis*, *C*. *subellipsoidea*, *D*. *salina*, *M*. *conductrix*, *P*. *renovo*, *P*. *soloecismus*, *Trebouxia sp*. *A1-2*, *V*. *carteri*) used in this study were downloaded from JGI’s PhycoCosm [91, 138–143]. Comparative genomic analysis was initiated by first inferring gene family relationships within and between the different species using OrthoFinder [51].

To visualize the distribution of gene families among the 17 species, a custom script was written to parse the assigned orthogroups from OrthoFinder and visualize the number and intersection of orthogroups by generating an UpSet plot. Similarly, the same script also generated weighted Venn diagrams for both the three *Botryococcus* species and the three *Chlamydomonas* species.

Gene gain and gene loss analysis was performed as previously described [61] with slight modifications. Briefly, the gene trees for each orthogroup from the OrthoFinder analysis were reconciled using Notung [60] to determine gene loss and gene gain over an evolutionary period. A custom script was written to extract these losses and gains at each node of the reconciled tree and mapped to the gene duplication species tree from the OrthoFinder analysis. The total number of organism gene duplication events was also obtained from the gene duplication specie trees.

### Identification of hydrocarbon genes within each *Botryococcus* species

Fatty acid elongase gene analysis was performed by by first obtaining FAE (Pfam: PF08392) and ELO (Pfam: PF01151) hidden-markov models (HMM) from Pfam [144, 145]. These HMM profiles were then used to search for homologous sequences using the HMMER suite [146] with an E-value cutoff of 10^-3^. Sequences that had significant homology to the FAE and ELO profiles in each *Botryococcus* species were then aligned using MAFFT and phylogeny was inferred with IQTREE using the same parameters mentioned above. KCR and HCD genes do not currently have a Pfam domain entry. Instead, single-copy protein sequences from *A*. *thaliana* were obtained from NCBI (NP_564905.1, and NP_193180.1 respectively) and used as query sequences in a blastp analysis of the *Botryococcus* species’ proteomes. An e-value cutoff of 10^-3^ was used. The Pfam ECR hmm domain (Pfam: PF12241) was used in a similar approach as the FAE and ELO searches. Due to the single-copy nature of KCR, HCD, and ECR genes, phylogenetic trees were not inferred. Squalene synthase (SS) and squalene synthase-like (SSL) gene analysis in the three *Botryococcus* species was performed in the same manner as the fatty acid elongases using the SQS-PYS HMM profile (Pfam: PF00494).

### Identification of meiosis genes within each *Botryococcus* species

Potential meiosis genes were identified based on sequence homology to meiosis genes identified in *C*. *subellipsoidea* and *C*. *variabilis* [79]. Briefly, DMC1, HOP1, HOP2, MER3, MND1, MSH4, MSH5, REC8, and SPO11 protein targets were identified in the BRAKER2 protein annotation sets by protein-protein BLAST+ homology alignment. Targets identified by alignment over an E-value threshold of 0.001, were then searched for Pfam domains using InterPro [144] against the Pfam database. In both homology searches, only the top alignment (smallest E-value) is reported. *C*. *subellipsoidea* protein sequences used in the homology searches were HOP1 (XP_005651810), HOP2 (XP_005643862), MER3 (XP_005651102, XP_005649357, XP_005649238), MND1 (XP_005649122), MSH4 (XP_005646103), MSH5 (XP_005644118), REC8 (XP_005651890), and SPO11 (XP_005647003). The *C*. *variabilis* meiosis gene used in the homology searches was DMC1 (XP_005848077).

## Supporting information

S1 File (PDF)
