# Supplementary material for: Reclassification of Botryococcus braunii chemical races into separate species based on a comparative genomics analysis
Source: PLoS One. 2024 Jul 29;19(7):e0304144. doi: 10.1371/journal.pone.0304144 (PMC11286282; doi:10.1371/journal.pone.0304144)
Supplement: S1 File — (PDF) [file pone.0304144.s001.pdf]

S1 Table. List of all species used in the comparative genomic analysis. All datasets were downloaded from PhycoCosm except for the *B. braunii* A and L races, which were generated in this study.

Species	Class	Strain	Genome Size (Mb)	Number of Genes	Author
Asterochlrois glomerata	Trebouxiophyceae	Cgr/DA1pho	55	10025	Armaleo *et al.*, 2019
Auxenochlorella protothecoides	Trebouxiophyceae	610	22	7013	Gao *et al.*, 2014
Botryococcus braunii, race A	Trebouxiophyceae	Yamanaka	188	18453	Boland *et al.*, 2024
Botryococcus braunii, race B	Trebouxiophyceae	Showa	170	23685	Browne *et al.*, 2017
Botryococcus braunii, race L	Trebouxiophyceae	Songkla Nakarin	135	15837	Boland *et al.*, 2024
Chlorella sorokiniana	Trebouxiophyceae		59	10384	Arriola *et al.*, 2018
Chlorella variabilis	Trebouxiophyceae	NC64A	46	9791	Blanc *et al.*, 2010
Coccomyxa subellipsoidea	Trebouxiophyceae	C-169	48	9629	Blanc *et al.*, 2012
Micractinium conductrix	Trebouxiophyceae	SAG 241.80	60	10070	Arriola *et al.*, 2018
Picochlorum renovo	Trebouxiophyceae		14	9010	Dahlin *et al.*, N/D
Picochlorum soloecismus	Trebouxiophyceae	DOE 101	15	6861	Gonzalez-Esquer *et al.*, 2018
Treboxia sp. A1-2	Trebouxiophyceae		52	13918	Greshake Tzovaras *et al.*, 2020
Volvox carteri	Chlorophyceae		131	13076	Prochnik *et al.*, 2010
Dunaliella salina	Chlorophyceae	CCAP 19/18	343	18801	Polle *et al.*, 2017
Chlamydomonas reinhardtii	Chlorophyceae		111	16403	Craig *et al.*, 2021
Chlamydomonas schloesseri	Chlorophyceae	UTEX 1602	130	16268	Craig *et al.*, 2021
Chlamydomonas incerta	Chlorophyceae	SAG 7.73	129	16957	Craig *et al.*, 2021

S2 Table. Comparative statistics of the OrthoFinder analysis.

Number of species	17
Number of genes	226181
Number of genes in orthogroups	200088
Number of unassigned genes	26093
Percentage of genes in orthogroups	88.5
Percentage of unassigned genes	11.5
Number of orthogroups	22024
Number of species-specific orthogroups	4617
Number of genes in species-specific orthogroups	17924
Percentage of genes in species-specific orthogroups	7.9
Mean orthogroup size	9.1
Median orthogroup size	5.0
G50 (assigned genes)	17
G50 (all genes)	15
O50 (assigned genes)	4121
O50 (all genes)	4939
Number of orthogroups with all species present	1652
Number of single-copy orthogroups	247

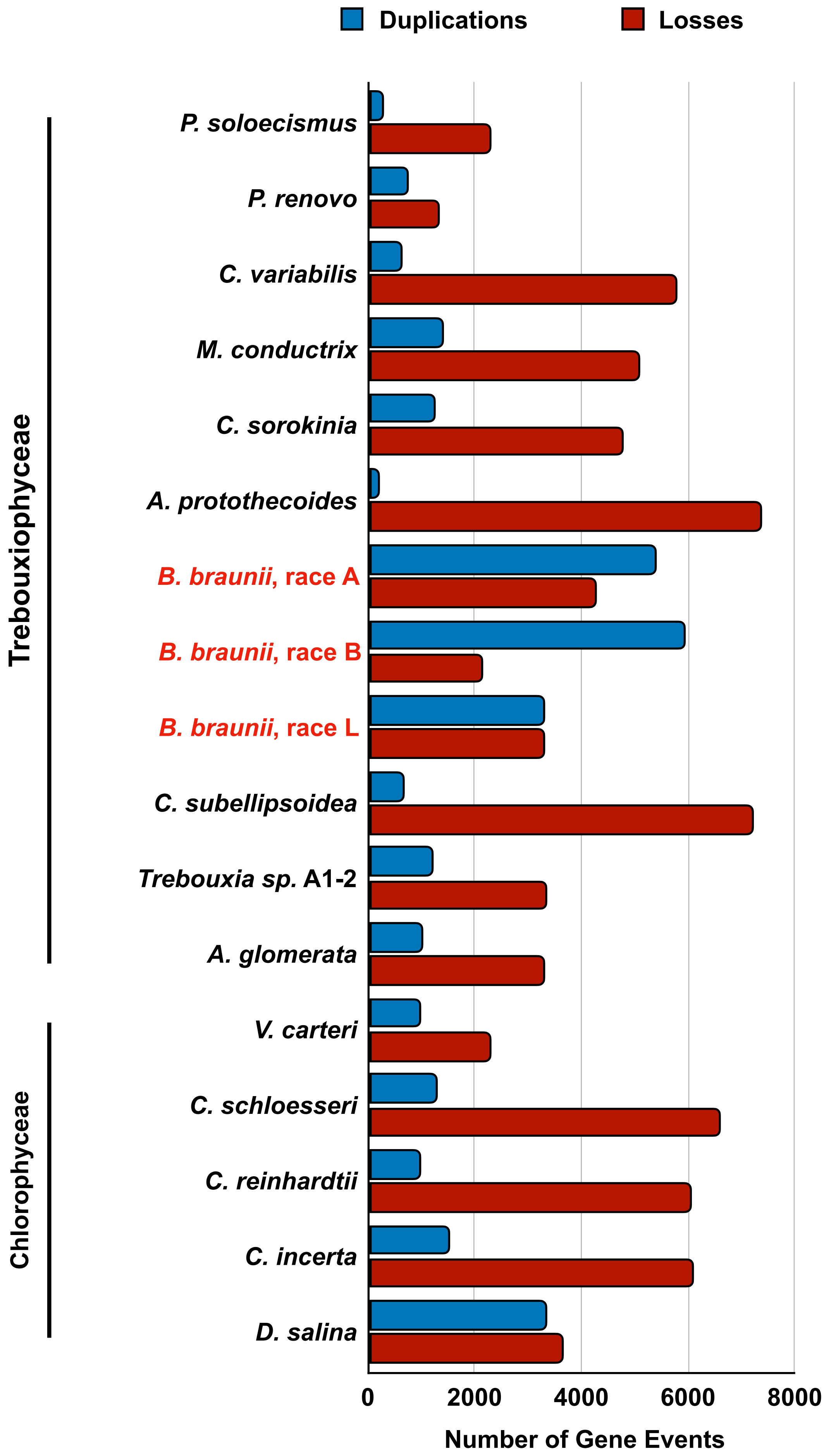

S1 Fig. Gene gain/loss analysis at each taxa break for Fig. 6B. Values shown here were used to generate the ratios in Fig. 6B by dividing the number of gains by the number of losses.

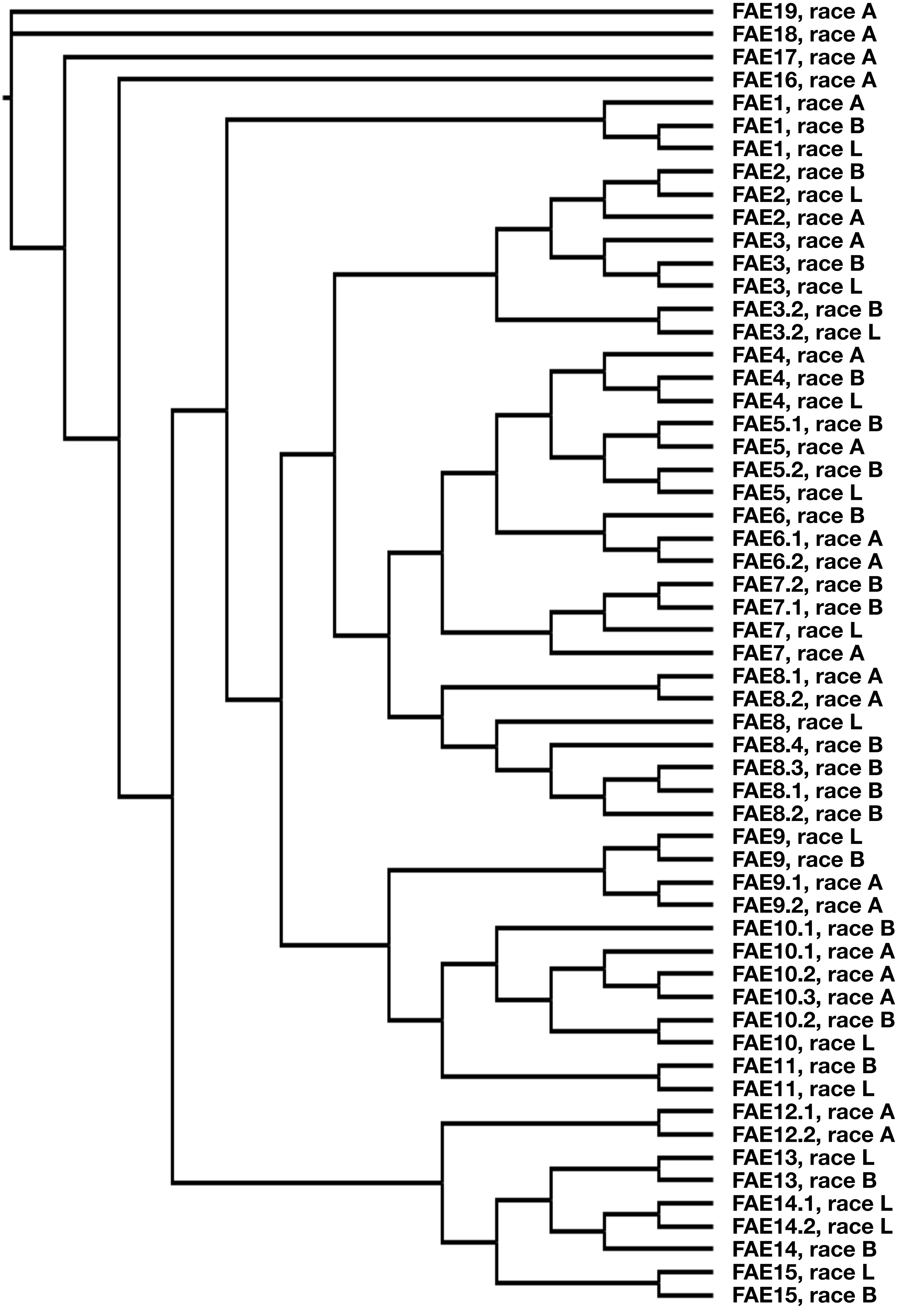

S2 Fig. Maximum likelihood phylogenetic inference of all FAE-like KCS genes in the three *B. braunii* races from the HMMER analysis using the Pfam ketoacyl-synt domain (Pfam: PF08392). The tree was visualized using FigTree with branch length normalized for equal clades. Genome assembly gene ID numbers for each gene identified can be found in S2 File.

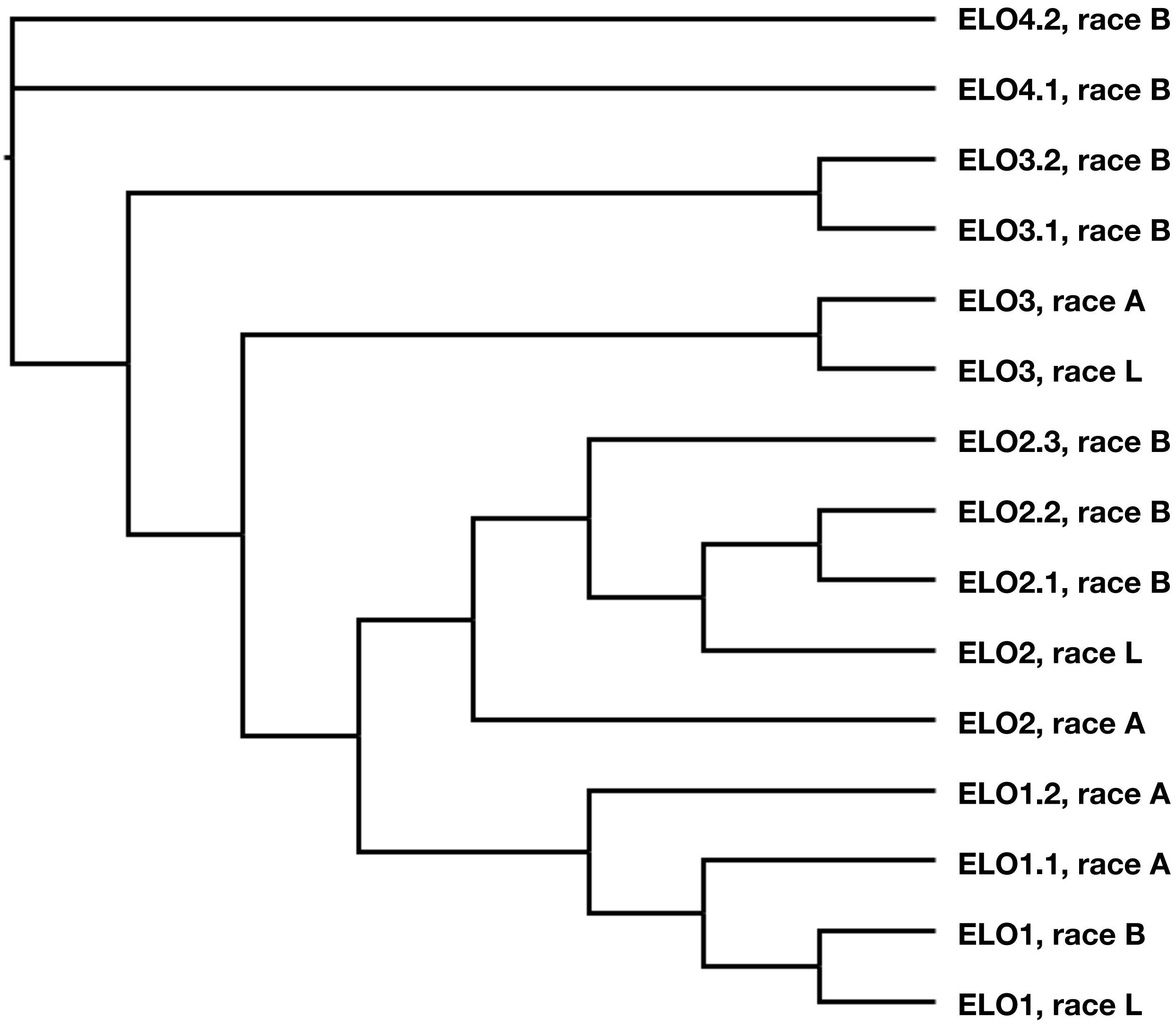

S3 Fig. Maximum likelihood phylogenetic inference of all ELO-like KCS genes in the three *B. braunii* races from the HMMER analysis using the Pfam ELO HMM profile (PF01151). The tree was visualized using FigTree with branch length normalized for equal clades. Genome assembly gene ID numbers for each gene identified can be found in S2 File.

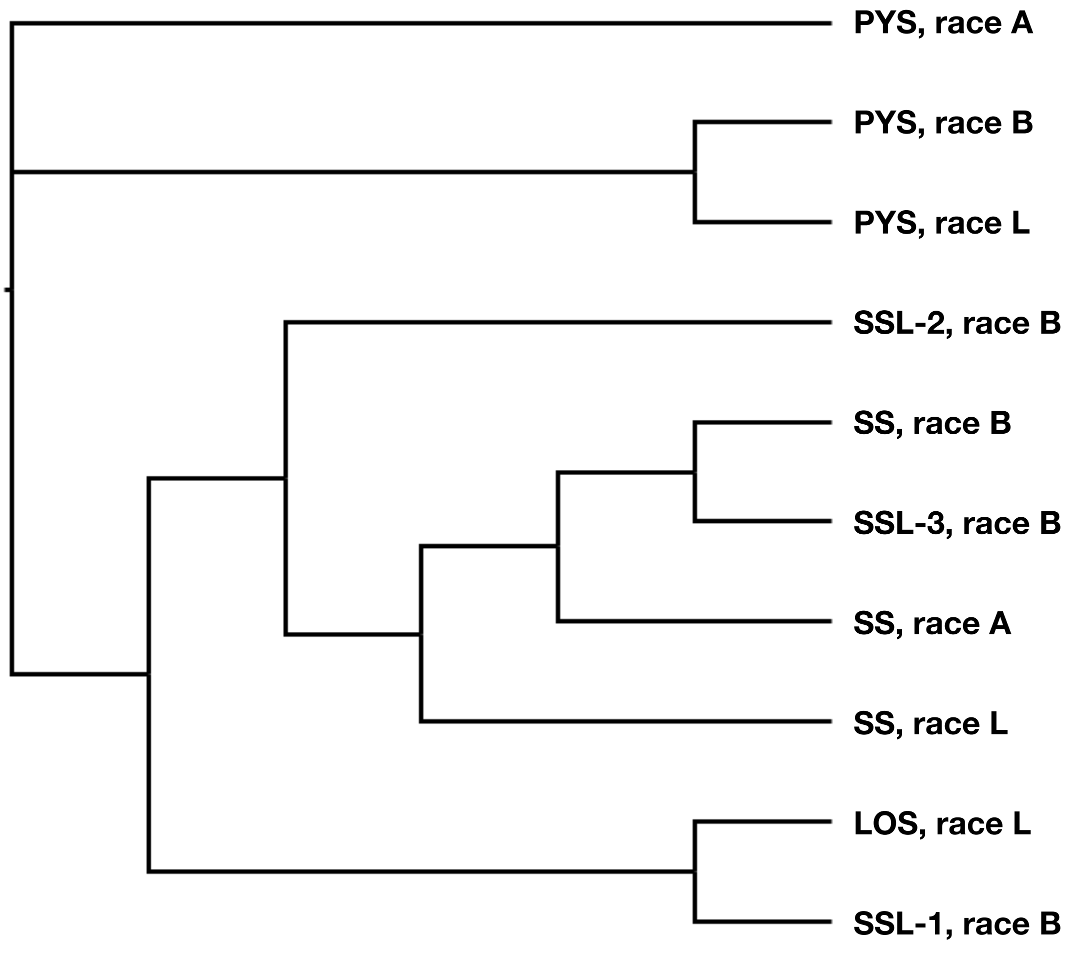

S4 Fig. Maximum likelihood phylogenetic inference of all SS, SS-like, and PSY genes in the three *B. braunii* races from the HMMER analysis using the Pfam SQS/PYS HMM profile (PF00494). The tree was visualized using FigTree with branch length normalized for equal clades. Genome assembly gene ID numbers for each gene identified can be found in S2 File.
